# Gap-closing experimental estimation of quantum Fisher information

**DOI:** 10.1126/sciadv.aef4342

**Published:** 2026-09-09

**Authors:** Qian-Xi Zhang, Qi-Ming Ding, Hu Chen, Shan Ling, Ya-Li Mao, Da-Jian Zhang, Xiao Yuan, Zheng-Da Li

**Affiliations:** ^1^ International Quantum Academy, Shenzhen 518048, China.; ^2^ Southern University of Science and Technology, Shenzhen 518055, China.; ^3^ Center on Frontiers of Computing Studies, Peking University, Beijing 100871, China.; ^4^ School of Computer Science, Peking University, Beijing 100871, China.; ^5^ School of Emerging Technology, University of Science and Technology of China, Hefei 230026, China.; ^6^ School of Physics, Nankai University, Tianjin 300071, China.; ^7^ Department of Physics, Shandong University, Jinan 250100, China.

## Abstract

Estimating the quantum Fisher information (QFI) is a central task in quantum science and technology, underpinning the benchmarking of measurement devices and the certification of metrological advantages. Owing to the highly nonlinear dependence of the QFI on the quantum state, achieving efficient estimates that reliably converge to the true value, which is a property known as gap closing, has remained a challenge. Here, we address it experimentally by implementing the recently proposed Krylov shadow tomography (KST) protocol on a versatile photonic platform capable of generating Greenberger-Horne-Zeilinger states of up to six qubits with controllable noise. By comparing KST with leading methods under matched resources, we show that it exhibits the predicted gap-closing behavior and yields a substantially lower mean absolute error. Moreover, we demonstrate that the resulting high-accuracy QFI estimates correctly predict the ultimate precision achievable in a phase estimation experiment, as dictated by the quantum Cramér-Rao bound. These results establish KST as a scalable and practical experimental tool for the efficient characterization and utilization of quantum resources in metrological applications.

## INTRODUCTION

The quantum Fisher information (QFI) is the cornerstone of quantum estimation theory (*1*, *2*), describing the ultimate measurement precision via the quantum Cramér-Rao bound (QCRB) (*3*–*7*). Ever since its inception half a century ago (*8*), the QFI has played a key role in quantum metrology and, more broadly, in quantum information science (*9*), with widespread applications such as characterization of metrologically useful entanglement (*10*–*24*), detection of critical phenomena in complex systems (*25*–*27*), assessment of trainability of quantum machine learning models (*28*), non-Hermitian quantum metrology (*29*), and factorization dynamics with quantum coherence (*30*).

However, a substantial challenge hinders the practical estimation of QFI: Its complex and nonlinear dependence on the quantum state renders the estimation task resource-intensive. Conventional protocols, particularly those requiring full state tomography, are experimentally infeasible for large-scale quantum systems because of the exponential growth of required resources. Existing alternative protocols (*31*–*40*) exhibit a stark trade-off: While computationally simple lower bounds are often too loose to be informative (*36*, *37*), more sophisticated approaches can be prohibitively expensive to guarantee convergence to the true QFI (*38*–*40*). To address this challenge, recent work proposed an estimation protocol based on Krylov shadow tomography (KST) (*41*, *42*). This hierarchical structure features a gap-closing property, converging progressively to the true QFI with tractable resources in relevant physical scenarios while remaining scalable to large systems. Although KST has emerged as a promising theoretical candidate for QFI estimation, its experimental viability and practical ability to be truly gap-closing remained a critical open question.

Here, we present an experimental benchmarking of the KST protocol for QFI estimation. We generate a series of Greenberger-Horne-Zeilinger (GHZ) states of up to six qubits under controlled noise by encoding qubits in both polarization and path degrees of freedom (DOFs) of four photons generated from two spontaneous parametric down-conversion (SPDC) sources. We show that the KST bound systematically converges to the true QFI and yields substantially lower mean absolute error than all existing prominent QFI estimation techniques, including sub-QFI bounds and state-of-the-art polynomial approximations based on Legendre and Taylor expansions, under strictly identical measurement resources. We further demonstrate its direct utility by providing a reliable bound on the achievable precision in a phase estimation task. Our results elevate KST from a theoretical proposal to an experimentally validated tool, establishing a powerful and practical protocol for characterizing and leveraging quantum resources.

## RESULTS

### KST protocol

Let ρ be a quantum state of *N* qubits. Equation 1 gives the QFI of ρ with respect to a Hermitian operator *H*

$$F_{Q}=2\sum_{k,l}\frac{{\left(\lambda_{k}-\lambda_{l}\right)}^{2}}{\lambda_{k}+\lambda_{l}}{|\langle k|H|l\rangle |}^{2}$$ (1)

where $\lambda_{k}$ and ∣k〉 represent the eigenvalues and eigenvectors of ρ, respectively, and we sum over all the indices *k* and *l* such that $\lambda_{k}+\lambda_{l}>0$ (*1*). Throughout this work, we set $H=\frac{1}{2}\sum_{j=1}^{N}\sigma_{z}^{\left(j\right)}$ unless we state otherwise, where $\sigma_{z}^{\left(j\right)}$ denotes the Pauli-*Z* operator acting on the *j*-th qubit. The KST protocol for estimating the QFI uses a Krylov subspace expansion technique to construct a nested sequence of subspaces (*41*). Within each subspace, the protocol formulates an optimal approximation to the QFI, resulting in a strict hierarchy of nonpolynomial lower bounds

$$B_{1}^{\left(\text{Kry}\right)}<B_{2}^{\left(\text{Kry}\right)}<\cdots <B_{n^{*}}^{\left(\text{Kry}\right)}=F_{Q}$$ (2)

where $n^{*}$ denotes the highest order of the nonpolynomial bounds. In many scenarios, particularly when ρ is of low rank, $n^{*}$ remains small, ensuring the practical efficiency of this hierarchy (*42*).

In particular, the *n*-th bound in the hierarchy, $B_{n}^{\left(\text{Kry}\right)}$, is defined as

$$B_{n}^{\left(\text{Kry}\right)}=b_{n}^{T}A_{n}^{-1}b_{n}$$ (3)

Here, the Hankel matrix $A_{n}$ and the vector $b_{n}$ are given by

$$A_{n}=\left(\begin{array}{cccc} T_{1} & T_{2} & \cdots & T_{n}\\ T_{2} & T_{3} & \cdots & T_{n+1}\\ \vdots & \vdots & \ddots & \vdots \\ T_{n} & T_{n+1} & \cdots & T_{2n-1} \end{array}\right),\;b_{n}=\left(\begin{array}{c} T_{0}\\ T_{1}\\ \vdots \\ T_{n-1} \end{array}\right)$$ (4)

We define the elements $T_{k}$ as

$$T_{k}≔\text{tr}\left(i\left[\rho ,H\right]R_{\rho }^{k}(i[\rho ,H])\right)$$ (5)

where $R_{\rho }\left(X\right)≔\frac{1}{2}\left(\rho X+X\rho \right)$. The quantities $T_{k}$ for k=0,1,…,2n−1 are polynomials in the density matrix ρ. For example, $T_{0}=2\text{tr}$$\left(\rho^{2}H^{2}-\rho H\rho H\right)$ and $T_{1}=\text{tr}\left(\rho^{3}H^{2}-\rho^{2}H\rho H\right)$.

The KST method guarantees convergence to the exact QFI in a finite number of increments in order. By leveraging the efficiency of classical shadows (*43*–*47*), the KST constructs a hierarchy of efficiently computable lower bounds for QFI. In this work, we focus on the first-order bound where *n* = 1, namely, $B_{1}^{\text{Kry}}$, for the following two reasons. First, for the pseudopure state, the Krylov bound converges exactly at $n^{*}=1$, yielding the exact QFI directly from the first-order bound: $F_{Q}=B_{1}^{\text{Kry}}$. The proof of this exact match is provided in the supplemental materials of (*41*). Second, recent theoretical advancements demonstrate that KST outperforms other polynomial approaches without requiring a priori assumptions about the underlying quantum states (*42*). Low-order Krylov bounds converge exponentially to the exact QFI and achieve exactness for low-rank states frequently encountered in practical settings. Because $B_{1}^{\text{Kry}}$ captures the primary contribution and provides an accurate estimate of the QFI, this theoretical basis justifies our adoption of the simplified depolarizing noise model in this experimental study. We detail the corresponding technical procedures in Materials and Methods.

### Experimental setup

**Fig. 1.**
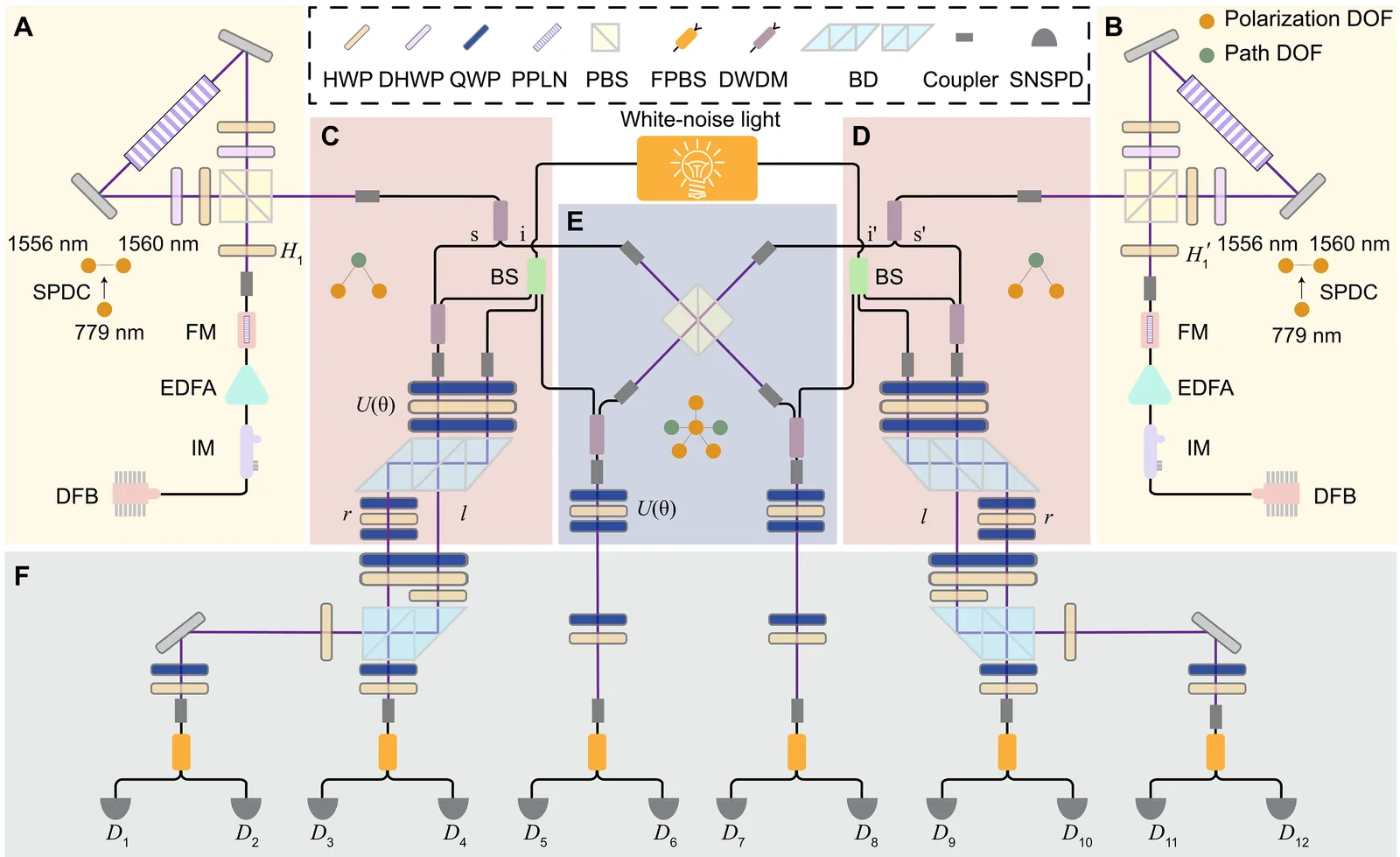
Schematic of the experimental setup. (**A** and **B**) Setups for preparing two-photon entangled states. (**C** and **D**) Setups for introducing path DOF, thereby generating two hyperentangled three-qubit quantum states. (**E**) Fusion of the two three-qubit states into a single six-qubit state. (**F**) Setup of measurements. DFB, distributed feedback laser; IM, intensity modulator; FM, frequency multiplier; EDFA, erbium-doped fiber amplifier; DPBS, dual-wavelength PBS; DHWP, HWP for dual wavelengths; PPLN, periodically poled MgO-doped lithium niobate; DWDM, dense wavelength division multiplexer; SNSPD, superconducting nanowire single-photon detector.

Experimentally, we construct a hybrid photonic quantum system to generate a specific type of pseudopure state, known as generalized Werner states, defined as $\rho_{N}\left(p\right)=\left(1-p\right)|{\text{GHZ}}_{N}\rangle \langle {\text{GHZ}}_{N}|+p\frac{I}{2^{N}}$, with up to six qubits encoded in polarization and path DOFs of photons. Here, $|{\text{GHZ}}_{N}\rangle =\left({|0\rangle }^{⊗N}+{|1\rangle }^{⊗N}\right)/\sqrt{2}$ represents the *N*-qubit GHZ state for *N* ≥ 3 and the Bell state for *N* = 2. Figure 1 schematically illustrates the experimental setup. Here, we focus on the detailed procedure for generating the six-qubit generalized Werner states.

First, as Fig. 1A shows, a laboratory-assembled laser module comprising a distributed feedback (DFB) laser, an intensity modulator (IM) driven by a pulse pattern generator, an erbium-doped fiber amplifier (EDFA), and a frequency multiplier (FM) generates a sequence of laser pulses with a duration of 66 ps and a central wavelength of 779 nm. By setting the half-wave plate (HWP) *H*_1_ at an angle α = 22.5°, we prepare each pulse in the state $\frac{1}{\sqrt{2}}\left(|H\rangle +|V\rangle \right)$, where *H* and *V* denote horizontal and vertical polarizations, respectively. We then bidirectionally inject these pump photons into a Sagnac interferometer containing a periodically poled MgO (magnesium oxide)–doped lithium niobate (PPLN) crystal, enabling type 0 SPDC (*48*, *49*). This process produces polarization-entangled photon pairs at 1556 nm for the signal and 1560 nm for the idler, and a dense wavelength division multiplexer (DWDM) spectrally separates them. Then, we generate a two-photon polarization-encoded Bell state $|{\text{GHZ}}_{2}\rangle =\frac{1}{\sqrt{2}}\left(|H_{s}H_{i}\rangle +|V_{s}V_{i}\rangle \right)$ with the subscripts s and i denoting signal and idler photons, respectively. We maintain the photon-pair production rate of the entanglement source at ∼0.04 per trigger.

Next, we direct the signal photons into a beam displacer (BD) to introduce an extra qubit encoded in the path DOF. We construct the BDs by bonding a polarizing beam splitter (PBS) with mirrors, which transmit photons in ∣H〉 polarization and reflect those in ∣V〉 polarization. After the photons pass through the BD, the BD transforms the state into a three-qubit GHZ state $|{\text{GHZ}}_{3}\rangle =\frac{1}{\sqrt{2}}\left(|H_{s}H_{i}l_{s}\rangle +|V_{s}V_{i}r_{s}\rangle \right)$, where $|l_{s}\rangle$ and $|r_{s}\rangle$ denote the left and right paths of the signal photons, respectively, as Fig. 1C illustrates. By using an identical setup, we further prepare a second copy of the hyperentangled three-qubit GHZ state $|\text{GH}\rangle Z_{3}^{\prime }\frac{1}{\sqrt{2}}\left(|H_{s^{\prime }}H_{i^{\prime }}l_{s^{\prime }}\rangle +|V_{s^{\prime }}V_{i^{\prime }}r_{s^{\prime }}\rangle \right)$, as Fig. 1 (B and D) shows.

Subsequently, we interfere the two idler photons on a spatial PBS to fuse the two three-qubit GHZ states as Fig. 1E shows. After applying postselection (*50*–*52*), we obtain a hyperentangled six-qubit GHZ state encoded in polarization and path DOFs across four photons

$$|{\text{GHZ}}_{6}\rangle =\frac{1}{\sqrt{2}}\left(|H_{s}H_{i}l_{s}H_{s^{\prime }}H_{i^{\prime }}l_{s^{\prime }}\rangle +|V_{s}V_{i}r_{s}V_{s^{\prime }}V_{i^{\prime }}r_{s^{\prime }}\rangle \right)$$ (6)

To introduce the white noise in the generalized Werner states, we use a modulated amplified spontaneous emission source followed by an unbalanced interferometer with a length difference of 2 m, thereby generating a sequence of fully depolarized photon pulses centered at 1558 nm. Then, we evenly split the photons into six parts using a fiber beam splitter and inject them into six distinct locations in the photonic network, resulting in a six-qubit maximally mixed state $\frac{I}{64}$. By generating the experimentally prepared GHZ state and the experimentally prepared maximally mixed state with time durations proportional to the desired probability *p* and recording the corresponding measurement outcomes, we effectively realize a six-qubit generalized Werner state with a tunable weight *p*

$$\rho_{6}\left(p\right)=\left(1-p\right)|{\text{GHZ}}_{6}\rangle \langle {\text{GHZ}}_{6}|+p\frac{I}{64}$$ (7)

Figure 1F illustrates the measurement setup. For photons carrying a single qubit encoded in the polarization DOF, we perform measurements using a combination of a quarter-wave plate (QWP) and an HWP placed before a fiber-polarizing beam splitter (FPBS). For photons hosting two qubits, one in the polarization DOF and the other in the path DOF, we measure the polarization qubit using a QWP and an HWP positioned before a BD, and we measure the path qubit using an additional set of a QWP and an HWP placed after the BD and before FPBSs. Superconducting nanowire single-photon detectors (SNSPDs) record all measurement outcomes as response events.

In our experiment, higher-order photon emissions in the SPDC sources, imperfect multiphoton interference at the PBS, and path-mode mismatch and interferometric instability when encoding qubits with BDs mainly cause the deviations from the ideal generalized Werner state. We provide further details in Supplementary Materials section D.2.

For two- to five-qubit states, the generation processes are analogous but technically more straightforward. The experimental setups are depicted in figs. S1 to S5. We characterized the produced GHZ states, with their fidelities reported in fig. S13 and table S2. Detailed state tomography results for these GHZ states are presented in figs. S14 to S18.

### Estimation of QFI via KST

**Fig. 2.**
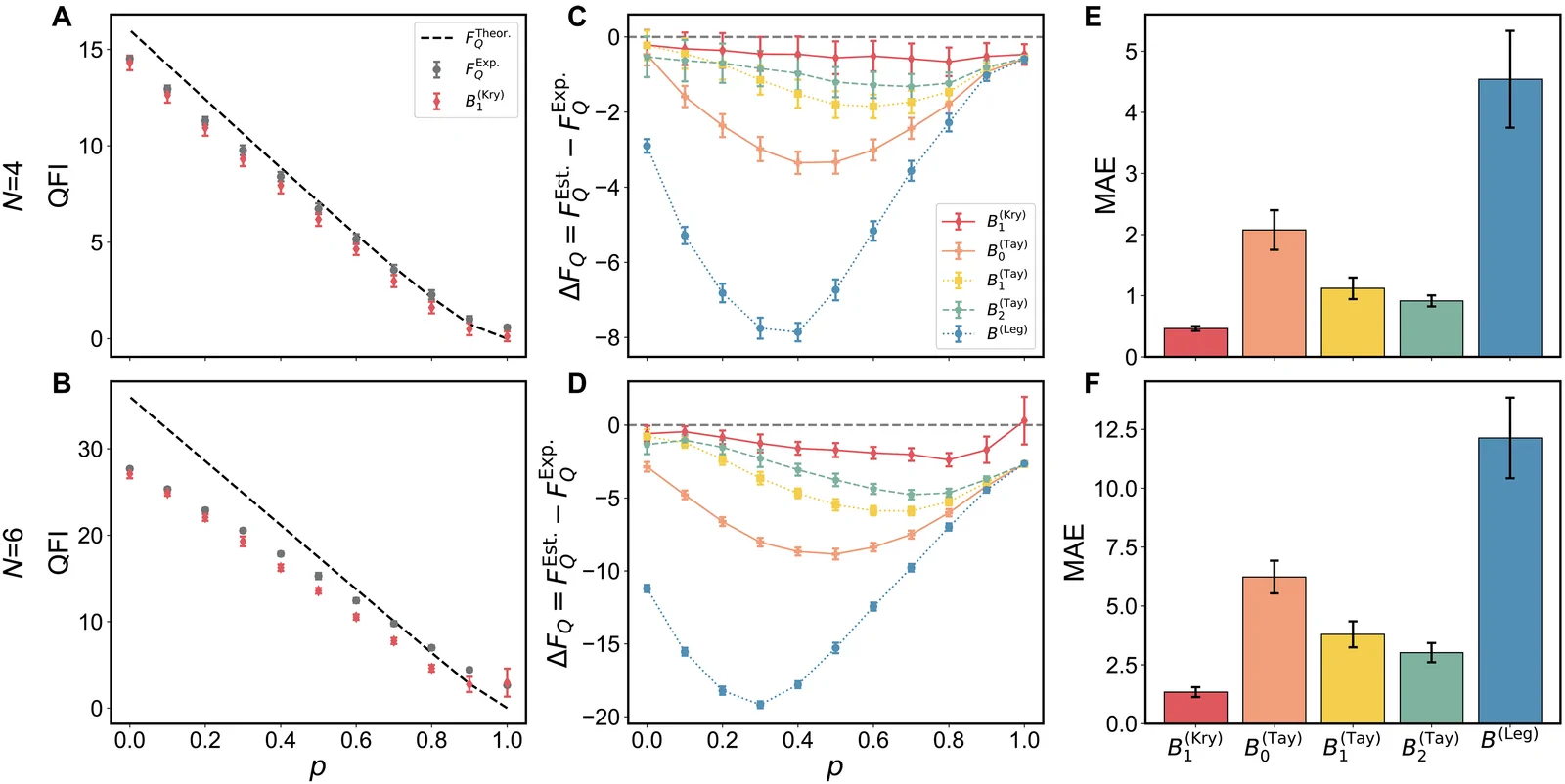
Evaluation of QFI estimation protocols for noisy GHZ states under varying white noise. Experimental and numerical results are partitioned into rows for *N* = 4 (top row) and *N* = 6 (bottom row) systems as a function of the noise parameter *p*. (**A** and **B**) Comparison of the QFI estimated via the KST protocol ($B_{1}^{\left(\text{Kry}\right)}$, red diamonds) against the theoretical baseline ($F_{Q}^{\text{Theor}.}$, dashed black line) and experimental quantum state tomography results ($F_{Q}^{\text{Exp}.}$, gray curve). (**C** and **D**) Deviation of the estimated QFI from the experimental tomography baseline ($F_{Q}^{\text{Exp}.}$) for the KST protocol. (**E** and **F**) Statistical performance comparison showing the mean absolute error (MAE) averaged over all *p* values for the KST (red), Taylor-based $B_{0}^{\left(\text{Tay}\right)}$ (orange), $B_{1}^{\left(\text{Tay}\right)}$ (yellow), $B_{2}^{\left(\text{Tay}\right)}$ (green), and Legendre (blue) protocols. Error bars represent standard deviations derived from 300 iterations of Poissonian resampling for experimental data or 100 rounds of random sampling for classical shadow–based estimators.

To perform a definitive, head-to-head comparison, we benchmark the prevailing protocols, including bounds based on the Legendre transform, $B^{\left(\text{Leg}\right)}$ (*38*), and a family of polynomial approximations derived from a Taylor series, such as the sub-QFI $B^{\left(\text{Sub}\right)}$ and its higher-order extensions $B_{n}^{\left(\text{Tay}\right)}$ (*36*, *39*), against our KST protocol $B_{1}^{\left(\text{Kry}\right)}$. We note that the sub-QFI $B^{\left(\text{Sub}\right)}$ is numerically equivalent to the zeroth-order Taylor expansion $B_{0}^{\left(\text{Tay}\right)}$. Therefore, we only present the zeroth-order Taylor expansion in the subsequent discussions and results, omitting the sub-QFI notation unless we specify otherwise. We further ensure a fair comparison under identical resource constraints by using the classical shadow formalism to compute all estimators from a single dataset. In particular, for each *N*-qubit system, we collected 1000 measurement shots for each of the $3^{N}$ Pauli bases. We then computed the estimators using *U*-statistics (*44*) and determined the error bars by calculating the standard deviation from random resampling. Figure 2 presents the results for *N* = 4 and *N* = 6 as representative cases; we provide comprehensive data for *N* = 2, 3, and 5, which support the same conclusions, in Supplementary Materials section C.6.

The results, presented in Fig. 2, demonstrate a clear hierarchy in performance. The KST estimator $B_{1}^{\left(\text{Kry}\right)}$ shows excellent agreement with the QFI value, $F_{Q}^{\text{Exp}.}$, which we obtained from full quantum state tomography for both *N* = 4 and *N* = 6 systems. An analysis of the deviation from tomography in Fig. 2 (C and D) and the mean absolute error in Fig. 2 (E and F) confirms this superiority. Across the entire noise range, $B_{1}^{\left(\text{Kry}\right)}$ is substantially more accurate than all competing approaches that we evaluated with the same resources.

Notably, while the KST protocol performs exceptionally well, we observe a small deviation from the benchmark value $F_{Q}^{\text{Exp}.}$ in Fig. 2 (C and D), indicating that the protocol does not perfectly close the gap in practice. Two primary reasons explain this deviation. First, the benchmark itself, derived from full quantum state tomography, often relies on a biased reconstruction method. Second, instabilities arise during the experimental acquisition of classical shadows because of environmental noise.

### Applications in quantum metrology

We further provide functional evidence of KST’s gap-closing capability beyond direct comparison to state tomography by benchmarking the QFI values that we estimate via KST against those that we infer from a standard phase estimation experiment. This scenario gives a clear and well-understood connection between measurement precision and the QFI, and we accomplish this by applying a unitary operation $U\left(\theta \right)=\text{diag}\left(1,e^{i\theta }\right)$ to each qubit of the experimentally prepared *N*-qubit GHZ states. Here, we introduce the unitary operation *U*(θ) by an HWP@θ/4 sandwiched between two QWP@45°, which transforms the GHZ state into $|\psi_{\theta }\rangle =\left({|0\rangle }^{⊗N}+e^{\text{iN}\theta }{|1\rangle }^{⊗N}\right)/\sqrt{2}$. For this state, the local measurement, $M=\sigma_{x}^{⊗N}$, is optimal and can saturate the QCRB, achieving an *N*-fold enhancement in phase sensitivity. We clarify, however, that such a QCRB-saturating local measurement generally does not exist for full-rank entangled states. Consequently, although $M=\sigma_{x}^{⊗N}$ is optimal in the pure-state setting, we no longer expect it to remain optimal in the presence of noise. This observation motivates us to prepare a high-purity GHZ state, for which the local measurement may provide a faithful benchmark for the QFI that KST estimates.

We explore the inverse phase variance, (Δθ)^−2^, as a direct and reliable proxy for the true QFI, which we determine experimentally via error propagation

$${\left(\Delta \theta \right)}^{2}=\frac{{\left(\Delta M\right)}^{2}}{{|\partial_{\theta }\langle M\rangle |}^{2}}=\frac{1-{\langle M\rangle }^{2}}{{|\partial_{\theta }\langle M\rangle |}^{2}}$$

**Fig. 3.**
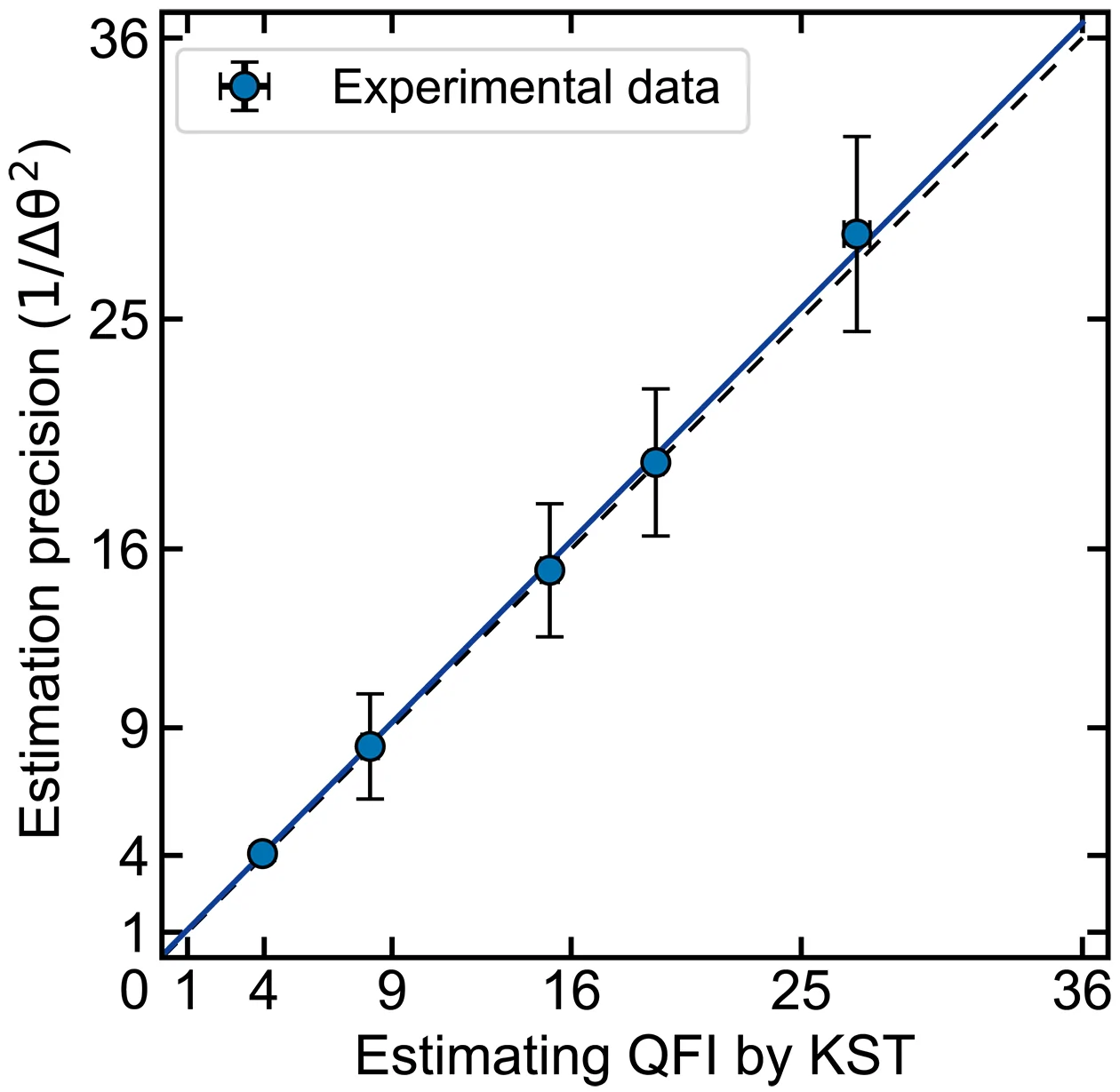
Linear relationship between the QFI estimated via the KST protocol and the inverse squared uncertainty [1/(Δθ)^2^] from a phase estimation experiment. The black dashed line represents the ideal reference line with a slope of 1 (*y* = *x*), and the blue solid line represents the weighted least-squares fit to the data (*y* = 1.01*x* + 0.08). The axes are plotted on an equidistant scale, although the displayed tick marks are nonequidistant.

We approximate the derivative ${\text{∂}}_{\theta }\langle M\rangle$ using the five-point stencil method (*53*). We provide details in Supplementary Materials section B.6. Figure 3 presents the central result of our work, directly comparing the QFI that our KST protocol estimates against this metrological benchmark, 1/(Δθ)^2^. By applying weighted least-squares fitting, we find that the data for all system sizes collapse onto a clear linear trend with a slope of 1.01. We provide fitting details in Supplementary Materials section E. The observed agreement indicates that the KST estimates are consistent with the metrological performance within experimental uncertainties.

Beyond validating KST, this result carries two further critical implications. First, it serves as a stringent, holistic benchmark of our entire experimental platform, confirming both the high fidelity of our GHZ state preparation and the near-optimality of our local measurement scheme. Second, it demonstrates the practical utility of the KST protocol as a diagnostic tool. It enables future experiments to efficiently characterize the metrological potential of complex quantum states without the prohibitive overhead of full state tomography, accelerating the development of next-generation quantum sensors.

## DISCUSSION

In summary, we present a comprehensive experimental validation of the KST protocol, demonstrating its capacity to provide accurate and resource-efficient estimates of the QFI in noisy multiqubit systems. Through a direct, head-to-head comparison under identical resource constraints, our work demonstrates the superiority of the KST protocol over other leading estimation techniques, including the sub-QFI and protocols based on Legendre or higher-order Taylor expansions. In addition, we confirm that the QFI estimates from KST reliably predict the ultimate metrological precision of a phase estimation task, thereby validating its practical utility and bridging the gap between theoretical bounds and experimental reality.

These results establish KST as a powerful and practical tool for the noisy, intermediate-scale quantum era, opening several exciting avenues for future work. Its efficiency makes it an ideal protocol for in situ characterization of multipartite entanglement in quantum simulators and for diagnosing the metrological value of states that quantum processors prepare, providing crucial feedback for hardware optimization. KST accelerates the exploration of fundamental many-body physics, enabling the experimental study of the QFI as a dynamic probe for quantum phase transitions (*25*–*27*), thermalization (*54*), and information scrambling in complex systems (*55*, *56*). By transforming the estimation of this key quantum resource from a prohibitive challenge into a practical one, KST paves the way for fully harnessing quantum systems for both fundamental discovery and next-generation sensing technologies.

## MATERIALS AND METHODS

### Robust estimation of *T_k_* via batch shadows

To estimate the multicopy observables *T_k_*, we implement the classical shadow formalism using randomized measurements (*44*). We apply random single-qubit Clifford unitaries to the quantum state ρ, followed by projective measurements in the computational basis. For each measurement shot, we construct an unbiased estimator of the density matrix by applying the inverse of the physical measurement channel to the resulting bitstrings.

We use *U*-statistics to construct unbiased estimators for the target functionals from these classical snapshots (*44*, *57*). While standard *U*-statistics can calculate any *c*-copy functional by summing over all combinations of *M* shadows, the classical postprocessing cost scales as $O\left(M^{c}\right)$. To resolve this scaling and mitigate the impact of statistical outliers, we use a batch shadow technique combined with a median-of-means estimator (*44*). We partition the *M* original snapshots into $K_{\text{total}}=K_{1}\times K_{2}$ equally sized subsets. Each subset *b* contains $M/K_{\text{total}}$ snapshots, which we average to construct a batch shadow ${\tilde{\rho }}^{\left(b\right)}$. The parameter $K_{2}$ determines the number of such batch shadows within each group, effectively controlling the trade-off between the precision of the *U*-statistics and the overall computational overhead.

We subsequently divide these $K_{\text{total}}$ batch shadows into $K_{1}$-independent groups. Within each group, we use the $K_{2}$ batch shadows to compute an unbiased *U*-statistic estimate for the target functional (*40*, *58*). Last, we determine the global estimate by calculating the median across all $K_{1}$ groups. We perform this median operation to suppress the influence of heavy-tailed statistical outliers and to guarantee that the estimates achieve a target accuracy ϵ with a bounded failure probability δ. This data processing pipeline reduces the time complexity from $O\left(M^{c}\right)$ to O(M). We provide the exact expressions for the permutation operators and the algorithmic pseudocode in Supplementary Materials sections C.1 and C.2 and table S1.

### Statistical analysis

To evaluate the data efficiency of the KST protocol, we collected 1000 measurement shots for each of the 3*^N^* Pauli bases across *N*-qubit states (N∈{2,3,4,5,6}). While full quantum state tomography uses the entire dataset, the classical shadow estimators in KST are restricted to random subsets spanning 10 to 80% of the total measurements. The statistical fluctuations of these estimators and the QFI are evaluated using a nonparametric bootstrap procedure (*59*). In this implementation, the experimental measurement outcomes are treated as independent and identically distributed samples, from which resampling with replacement is subsequently performed. This represents a rudimentary variant of the bootstrap method. Realistic experimental data inevitably exhibit nonstationary and nonideal properties resulting from system drifts. While block bootstrapping can theoretically accommodate short-term local correlations, it remains inadequate for global long-term drifts (*60*, *61*). Rigorously addressing these experimental features would require more sophisticated statistical techniques, such as residual bootstrapping and subsampling for weak correlations (*62*). We clarify that implementing these advanced methods alongside a rigorous consistency validation is, unfortunately, currently unfeasible. The substantial classical computational overhead required to process the massive number of higher-order batch shadow combinations for our evaluated systems, particularly for the six-qubit system, constrained our procedure to 100 independent replications. The phase variance (Δθ)^2^ is extracted via the five-point stencil method (*53*), with its uncertainty derived from synthetic datasets generated under the assumption of Poissonian photon counting statistics. Details regarding the bootstrap procedure, the five-point stencil implementation, and source count rates are provided in Supplementary Materials section B.7.

## Supplementary Materials

### This PDF file includes:

Supplementary Text Figs. S1 to S18 Tables S1 and S2 References
