# Supplementary material for: Gap-closing experimental estimation of quantum Fisher information
Source: Sci Adv. 2026 Sep 9;12(37):eaef4342. doi: 10.1126/sciadv.aef4342 (PMC13557076; doi:10.1126/sciadv.aef4342)
Supplement: Supplementary file 1 [file sciadv.aef4342_sm.pdf]

Science Advances
AAAS

Supplementary Materials for

Gap-closing experimental estimation of quantum Fisher information

Qian-Xi Zhang *et al.*

Corresponding author: Da-Jian Zhang, zdj@sdu.edu.cn; Xiao Yuan, xiaoyuan@pku.edu.cn;
Zheng-Da Li, lizhengda@iqasz.cn

Sci. Adv. **12**, eaef4342 (2026)
DOI: 10.1126/sciadv.aef4342

This PDF file includes:

Supplementary Text
Figs. S1 to S18
Tables S1 and S2
References

A Previous theoretical work on estimating QFI

A.1 Legendre Transform Bound

Reference (*38*) proposed a lower bound derived from the Legendre transform of F_Q. Equation (S1) defines this bound as

$$B^{(\text{Leg})} = \begin{cases} N^2(1-2f_{\text{GHZ}})^2 & \text{if } f_{\text{GHZ}} > \frac{1}{2}, \\ 0 & \text{if } f_{\text{GHZ}} \leq \frac{1}{2}, \end{cases} \tag{S1}$$

where $f_{\text{GHZ}} = \langle \text{GHZ}|\rho|\text{GHZ}\rangle$ denotes the fidelity between the state ρ and the target GHZ state.

A.2 Taylor Expansion Bounds

Reference (*39*) derived an infinite series of polynomial lower bounds from a Taylor expansion of F_Q. Equation (S2) provides the general form for the n-th order bound:

$$B_n^{(\text{Tay})} = 2\,\text{Tr}\sum_{l=0}^{n}\left((\rho\otimes\mathbb{I}-\mathbb{I}\otimes\rho)^2 \times (\mathbb{I}\otimes\mathbb{I}-\rho\otimes\mathbb{I}-\mathbb{I}\otimes\rho)^l \times S(H\otimes H)\right) \tag{S2}$$

where S is the swap operator and $\mathbb{I}$ is the identity operator.

For the specific orders $n = 0, 1$, and 2 that we use in our benchmark, Reference (*36*) initially introduced the 0th-order term based on the concept of the sub-QFI. Equation (S3) gives this 0th-order bound directly:

$$B_0^{(\text{Tay})} = -2\,\text{Tr}\left([\rho, H]^2\right) = 4\,\text{Tr}(\rho^2H^2 - \rho H\rho H), \tag{S3}$$

where the square brackets denote the commutator. Equations (S4) and (S5) define the first-order and second-order bounds:

$$B_1^{(\text{Tay})} = 2B_0^{(\text{Tay})} - 4\,\text{Tr}(\rho^2[\rho, H]H), \tag{S4}$$

$$B_2^{(\text{Tay})} = 3(B_1^{(\text{Tay})} - B_0^{(\text{Tay})}) + 4\,\text{Tr}(\rho^2[\rho^2, H]H). \tag{S5}$$

While $B_n^{(\mathrm{Tay})}$ converges to F_Q as $n \to \infty$, the experimental resources that we require to estimate each subsequent term grow exponentially. This exponential growth makes the protocol impractical for large n.

B The details of experiment

B.1 Entanglement source

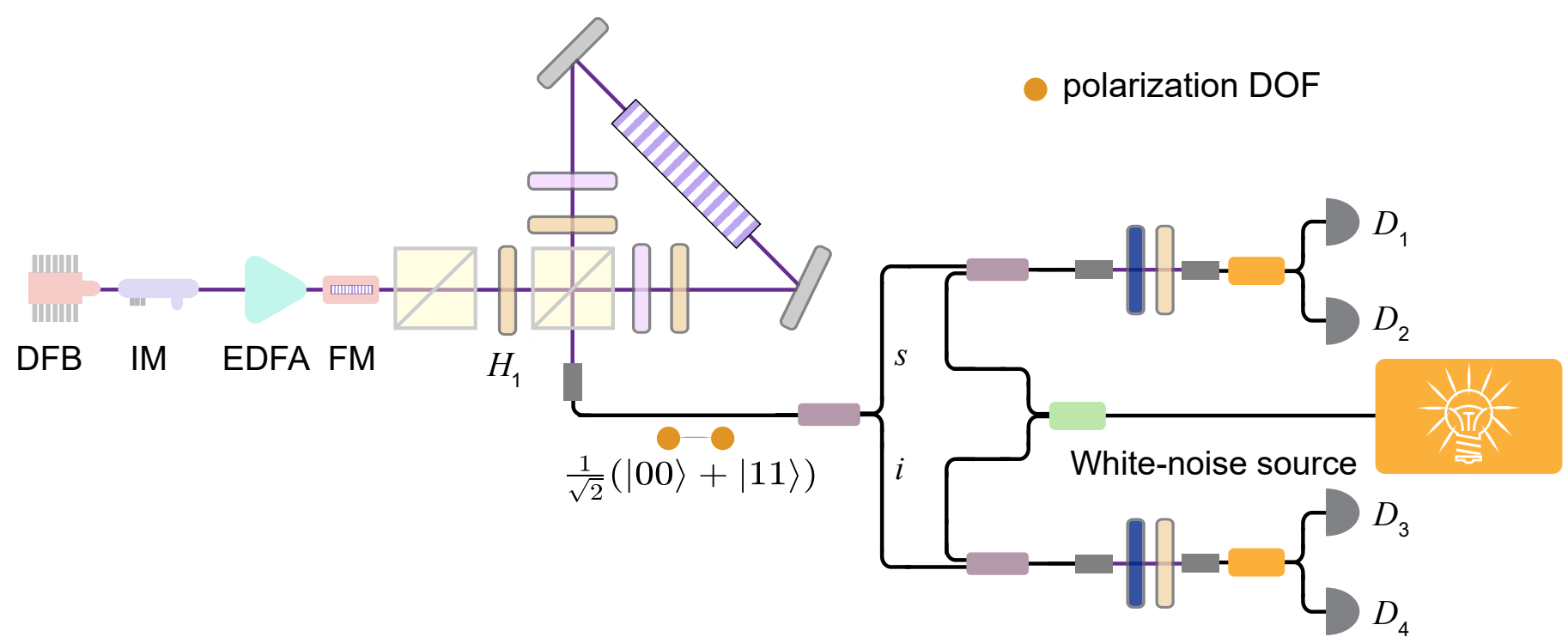

Figure S1: **Experimental setup of the 2-qubit states.**

As Fig. S1 illustrates, a distributed feedback (DFB) laser generates continuous-wave (CW) light at a central wavelength of 1558 nm. A pulse pattern generator (PPG) drives an intensity modulator (IM) with a 100 MHz trigger signal. The IM modulates this CW light, producing optical pulses with a temporal width of 66 ps. An erbium-doped fiber amplifier (EDFA) then amplifies the pulses, and a frequency multiplier (FM) incorporating a periodically poled MgO-doped lithium niobate (PPLN) crystal frequency-doubles them to 779 nm. After a dense wavelength division multiplexer (DWDM) spectrally filters the light, the resulting 779 nm pulsed laser serves as the pump for the entangled photon pair source. By setting the half-wave plate (HWP) H_1 at an angle $\alpha = 22.5°$, we prepare each pulse in the polarization state $\frac{1}{\sqrt{2}}(|H\rangle + |V\rangle)$, where H and V denote horizontal and vertical polarizations, respectively.

The pulses then propagate bidirectionally through a PPLN crystal placed in a Sagnac interferometer, with horizontal and vertical components undergoing type-0 SPDC (*48, 49*). This process produces pairs of photons at 1556 nm for the signal and 1560 nm for the idler, and a DWDM filter spectrally separates them. The source generates the two-photon polarization state $|\phi\rangle = \frac{1}{\sqrt{2}}(|H_s H_i\rangle + |V_s V_i\rangle)$, with s and i labeling the signal and idler photons, respectively. After we spectrally filter the photons with a 3.3 GHz bandpass filter, the resulting entangled state achieves a fidelity exceeding 0.98. We maintain the photon-pair production rate of the entanglement source at approximately 0.007 per trigger.

To introduce the white noise in the generalized Werner states, we employ a modulated amplified spontaneous emission (ASE) source followed by an unbalanced interferometer with a length difference of 2 m, thereby generating a sequence of fully depolarized photon pulses centered at 1558 nm. Then, we evenly split the photons into two parts using a fiber beam splitter (FBS) and inject them into two distinct locations in the photonic network. Figure S1 illustrates this injection location. This process results in a 2-qubit maximally mixed state $\frac{\mathbb{I}}{4}$. By generating the experimentally prepared GHZ state and the experimentally prepared maximally mixed state with time durations proportional to the desired probability p, and recording the corresponding measurement outcomes, we effectively realize a two-qubit generalized Werner state with a tunable weight p:

$$\rho_2(p) = (1-p)|\phi\rangle\langle\phi| + p\frac{\mathbb{I}}{4}. \tag{S6}$$

B.2 Experimental setup of the 4-qubit states

The experimental setup for generating 4-qubit pseudopure states is illustrated in Fig. S2. For the pure-state component, a four-photon GHZ state $|\mathsf{GHZ}_4\rangle$ is prepared by combining two entangled sources. By interfering the two idler photons from each copy on a PBS and applying

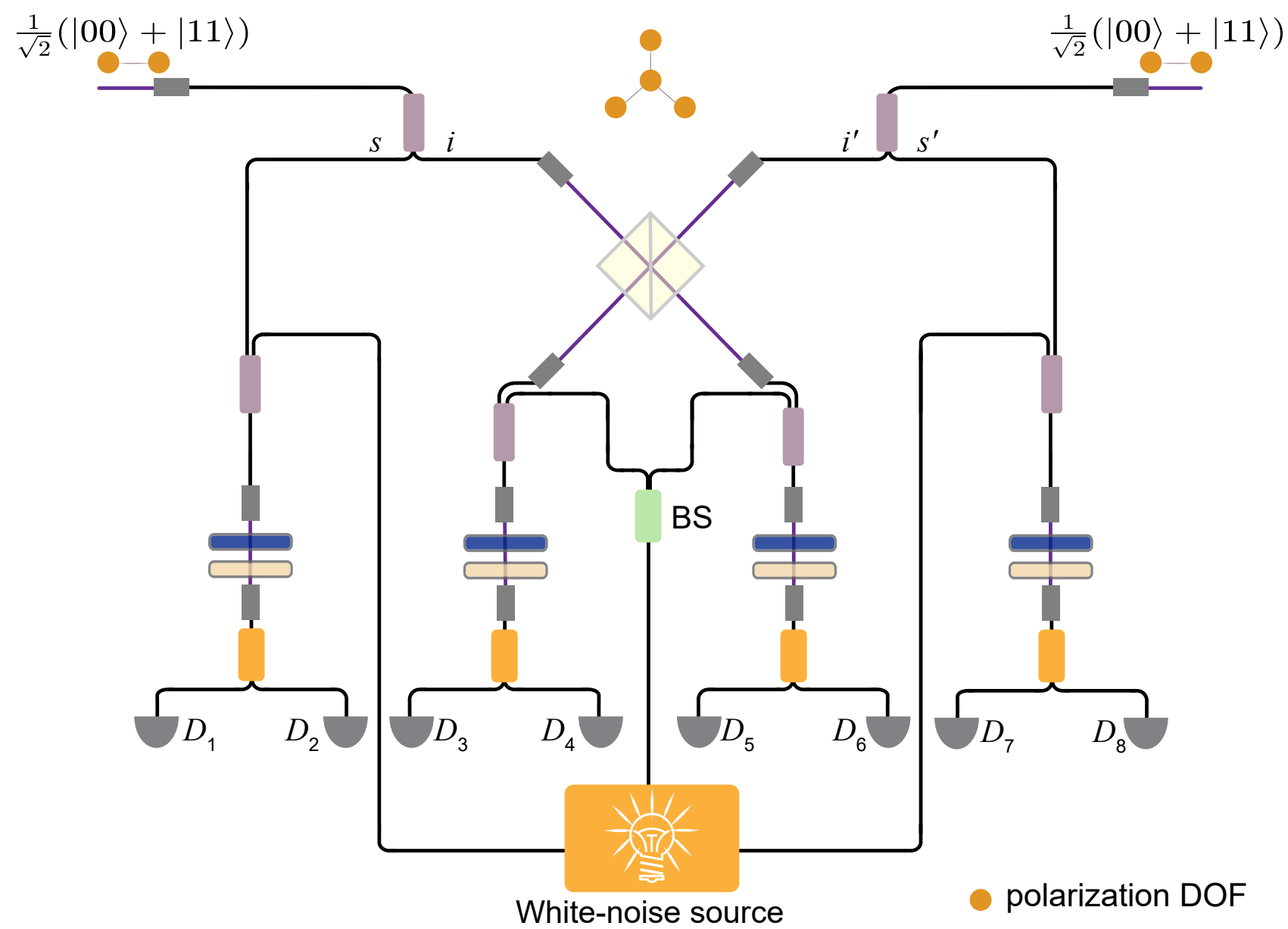

Figure S2: **Experimental setup of the 4-qubit states.**

post-selection (*50–52*), we obtain a four-photon GHZ state of the form

$$|\mathsf{GHZ}_4\rangle = \frac{1}{\sqrt{2}} \left(|H_s H_i H_{s'} H_{i'}\rangle + |V_s V_i V_{s'} V_{i'}\rangle \right), \tag{S7}$$

where the qubits are encoded in the polarization DOF. The photon-pair production rate of each entanglement source is maintained at approximately 0.007 per trigger.

To introduce the white noise in the generalized Werner states, we employ a modulated amplified spontaneous emission (ASE) source followed by an unbalanced interferometer with a length difference of 2 m, thereby generating a sequence of fully depolarized photon pulses centered at 1558 nm. Then the photons are evenly split into two parts using a fiber beam splitter (FBS) and injected into two distinct locations in the photonic network (see Fig. S2), resulting in 4-qubit maximally mixed state $\frac{\mathbb{I}}{16}$. By generating the experimentally prepared GHZ state and the experimentally prepared maximally mixed state with time durations proportional to the desired probability p, and recording the corresponding measurement outcomes, we effectively

realize a 4-qubit generalized Werner state with a tunable weight p,

$$\rho_4(p) = (1-p)|\mathsf{GHZ}_4\rangle\langle\mathsf{GHZ}_4| + p\frac{\mathbb{I}}{16}. \tag{S8}$$

B.3 Experimental setup of the 3-qubit states

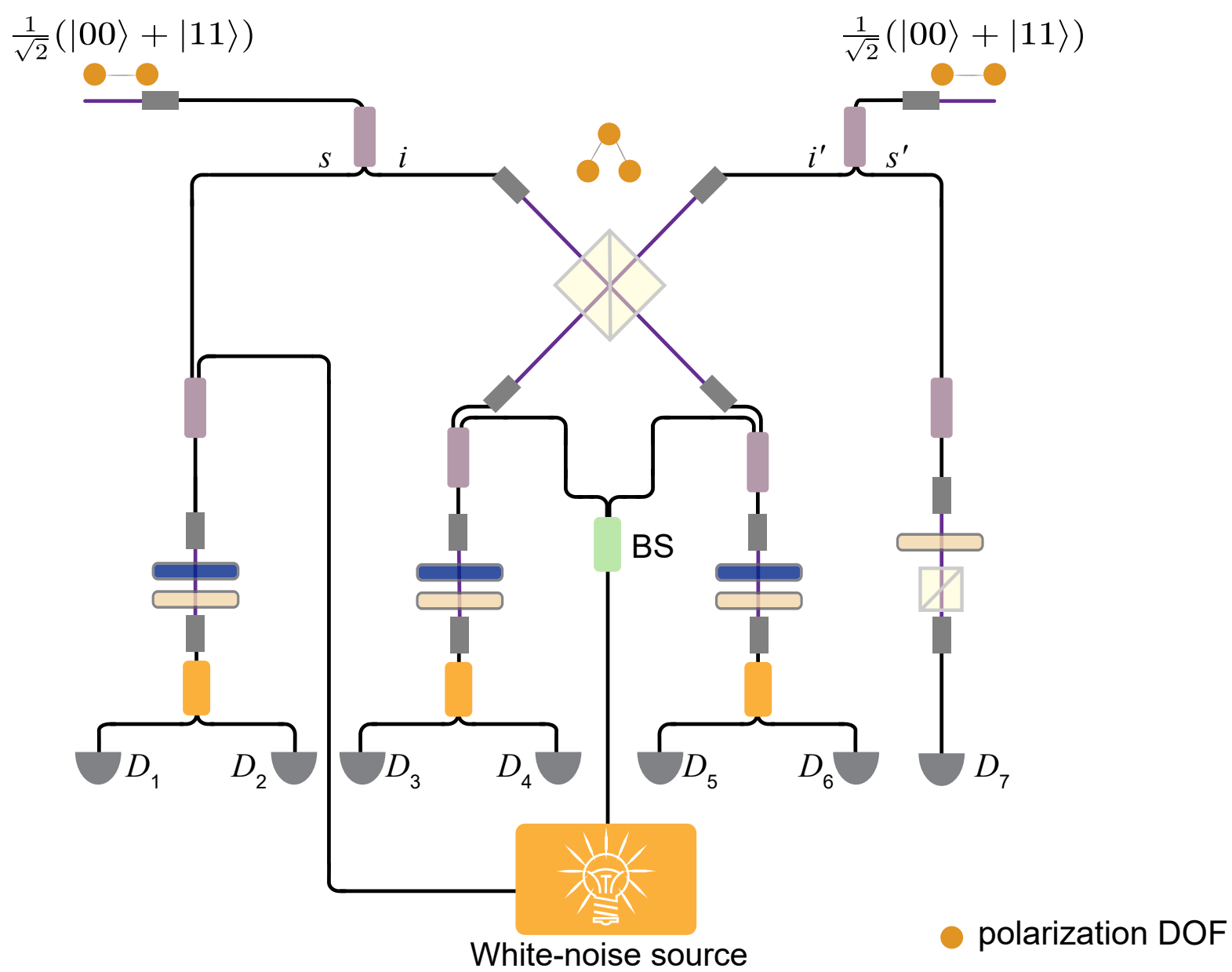

Figure S3: **Experimental setup of the 3-qubit states.**

The experimental setup for generating 3-qubit pseudopure states is illustrated in Fig. S3. Based on the four-photon GHZ state, projecting the heralding photon onto the $|+\rangle$ state collapses the remaining three photons into a three-photon GHZ state.

$$|\mathsf{GHZ}_3\rangle = \frac{1}{\sqrt{2}}\left(|H_s H_i H_{i'}\rangle + |V_s V_i V_{i'}\rangle\right), \tag{S9}$$

with all qubits encoded in the polarization degree of freedom (DOF). The photon-pair production rate of each entanglement source is maintained at approximately 0.007 per trigger.

To introduce the white noise in the generalized Werner states, we employ a modulated amplified spontaneous emission (ASE) source followed by an unbalanced interferometer with a

length difference of 2 m, thereby generating a sequence of fully depolarized photon pulses centered at 1558 nm. Then the photons are evenly split into two parts using a fiber beam splitter (FBS) and injected into two distinct locations in the photonic network (see Fig. S3), resulting in 3-qubit maximally mixed state $\frac{\mathbb{I}}{8}$. By generating the experimentally prepared GHZ state and the experimentally prepared maximally mixed state with time durations proportional to the desired probability p, and recording the corresponding measurement outcomes, we effectively realize a 3-qubit generalized Werner state with a tunable weight p,

$$\rho_3(p) = (1-p)|\mathsf{GHZ}_3\rangle\langle\mathsf{GHZ}_3| + p\frac{\mathbb{I}}{8}. \quad \text{(S10)}$$

B.4 Experimental setup of the 5-qubit states

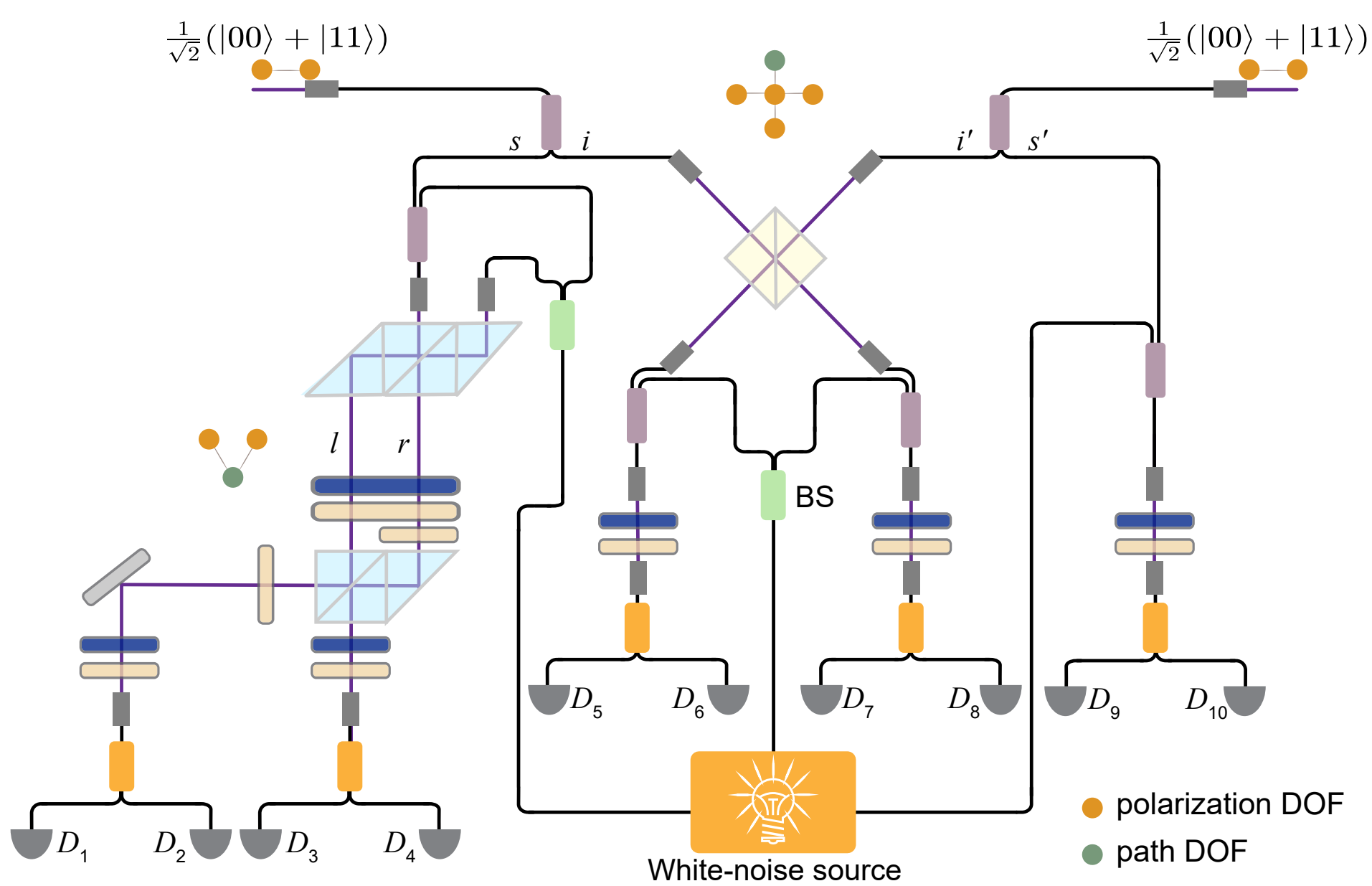

Figure S4: **Experimental setup of the 5-qubit states.**

The experimental setup for generating 5-qubit pseudopure states is illustrated in Fig. S4. For the pure-state component, the polarization-entangled photons are directed into an apparatus composed of beam displacer (BD) to introduce the path qubit and generate three-qubit quantum

states. The evolution of the states is shown as follows:

$$\frac{1}{\sqrt{2}}\left(|H_sH_i\rangle + |V_sV_i\rangle\right) \xrightarrow{\text{BD}} \frac{1}{\sqrt{2}}\left(|H_sH_il_s\rangle + |V_sV_ir_s\rangle\right), \tag{S11}$$

where $|l_s\rangle$ and $|r_s\rangle$ denote the upper and lower paths of the signal photon, respectively.

To construct the 5-qubit GHZ state, we first generate this hybrid-coded three-qubit state $\frac{1}{\sqrt{2}}\left(|H_sH_il_s\rangle + |V_sV_ir_s\rangle\right)$, with qubits encoded in both polarization and path DOFs. By interfering the idler photon from the hybrid-coded three-qubit state $\frac{1}{\sqrt{2}}\left(|H_sH_il_s\rangle + |V_sV_ir_s\rangle\right)$ with a two-photon entanglement state $|\phi\rangle$ on a PBS and applying post-selection, we obtain the 5-qubit GHZ state

$$|\mathsf{GHZ}_5\rangle = \frac{1}{\sqrt{2}}\left(|H_sH_il_sH_{s'}H_{i'}\rangle + |V_sV_ir_sV_{s'}V_{i'}\rangle\right), \tag{S12}$$

where the qubits are encoded in both polarization and path DOFs. The photon-pair production rate of each entanglement source is maintained at approximately 0.04 per trigger.

To introduce the white noise in the generalized Werner states, we employ a modulated amplified spontaneous emission (ASE) source followed by an unbalanced interferometer with a length difference of 2 m, thereby generating a sequence of fully depolarized photon pulses centered at 1558 nm. Then the photons are evenly split into two parts using a fiber beam splitter (FBS) and injected into two distinct locations in the photonic network (see Fig. S4), resulting in 5-qubit maximally mixed state $\frac{\mathbb{I}}{32}$. By generating the experimentally prepared GHZ state and the experimentally prepared maximally mixed state with time durations proportional to the desired probability p, and recording the corresponding measurement outcomes, we effectively realize a 5-qubit generalized Werner state with a tunable weight p,

$$\rho_5(p) = (1-p)|\mathsf{GHZ}_5\rangle\langle\mathsf{GHZ}_5| + p\frac{\mathbb{I}}{32}. \tag{S13}$$

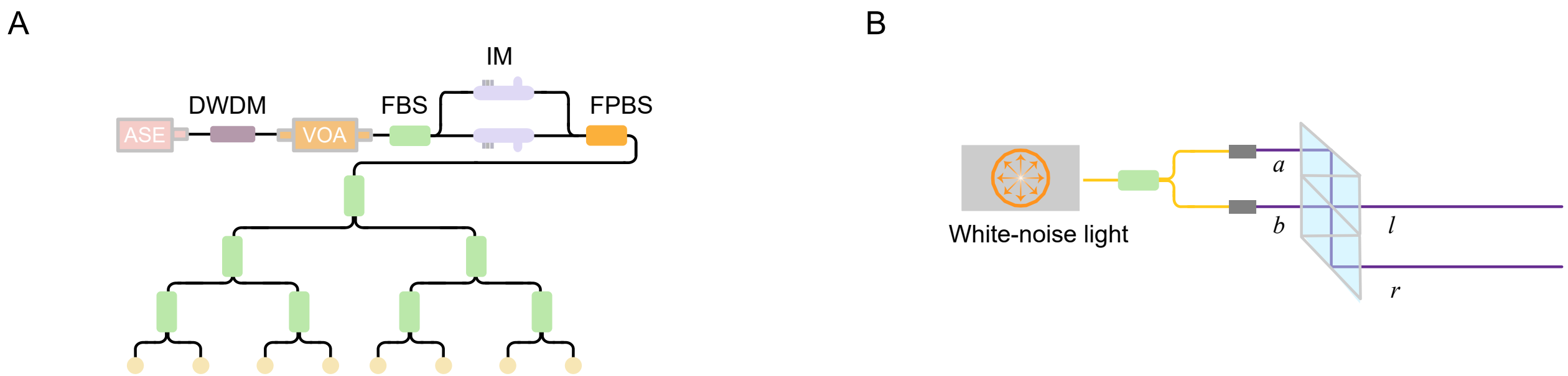

Figure S5: **Experimental configuration for generating noisy multiphoton states.** (A) Schematic of the white-noise source, illustrating the optical components and pathways used to produce controlled decoherence. (B) Detailed experimental setup for the generation and characterization of 5-qubit and 6-qubit hybrid-encoded states subjected to the white noise.

B.5 Experimental implementation of white noise source

The experimental setup of the white noise source is shown in Fig. S5A. The amplified spontaneous emission (ASE) source is filtered by a DWDM to retain the wavelength component at 1558 nm and then passes through a variable optical attenuator (VOA). The filtered light is subsequently modulated by an IM, driven by a 100 MHz trigger signal from a PPG, to produce optical pulses with a temporal width of 66 ps.

Since the IM introduces polarization dependence, the light is first split by a 50:50 fiber beam splitter (FBS) into two paths, each modulated independently by an IM, and then recombined at an FPBS. To enhance phase randomness, the setup employs an unbalanced interferometer with a path-length difference of 2 m. Finally, the modulated white noise pulses are evenly divided into eight parts by a cascaded FBS network and injected into the white-noise input ports indicated in Fig. 1 of the main text.

For the 5- to 6-qubit white noise state, the experimental setup to generate the white noise of two qubits, composed of a path qubit and its associated polarization qubit, is shown in Fig. S5 B. The photons entering from path a in the states $|H\rangle\langle H|$ and $|V\rangle\langle V|$ are transformed by BD_1

into $|Hr\rangle\langle Hr|$ and $|Vl\rangle\langle Vl|$, respectively. Similarly, photons entering from path b in the states $|H\rangle\langle H|$ and $|V\rangle\langle V|$ are transformed by BD_1 into $|Hl\rangle\langle Hl|$ and $|Vr\rangle\langle Vr|$, respectively. In this way, a two-qubit white-noise state of the form $\frac{1}{4}(|Hl\rangle\langle Hl|+|Hr\rangle\langle Hr|+|Vl\rangle\langle Vl|+|Vr\rangle\langle Vr|)$ is realized.

By generating the experimentally prepared GHZ state and the experimentally prepared maximally mixed state with time durations proportional to the desired probability p, and recording the corresponding measurement outcomes, we effectively realize an N-qubit generalized Werner state with a tunable weight p:

$$\rho_N(p) = (1-p)|\mathsf{GHZ}_N\rangle\langle\mathsf{GHZ}_N| + p\frac{\mathbb{I}}{2^N}, \tag{S14}$$

where $|\mathsf{GHZ}_2\rangle = |\phi\rangle$.

B.6 Experimental implementation of phase shift unitaries on GHZ States

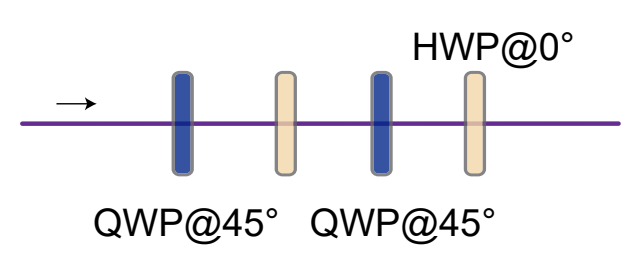

Figure S6: **Experimental setup of phase-shift unitaries.**

We implement the unitary operation

$$U = \begin{pmatrix} 1 & 0 \\ 0 & e^{i\theta} \end{pmatrix} \tag{S15}$$

on each qubit of the N-qubit GHZ state

$$|\mathrm{GHZ}_N\rangle = \frac{1}{\sqrt{2}}\left(|0\rangle^{\otimes N} + |1\rangle^{\otimes N}\right) \tag{S16}$$

using a wave-plate sequence consisting of an HWP at angle $\frac{\theta}{4}$, sandwiched between two QWPs at $45°$, followed by an HWP at $0°$ (see Fig. S6). In matrix notation, the sequence can be written

as

$$\begin{aligned} U &= \mathrm{HWP}(0^\circ) \cdot \mathrm{QWP}(45^\circ) \cdot \mathrm{HWP}(\frac{\theta}{4}) \cdot \mathrm{QWP}(45^\circ) \\ &= \begin{pmatrix} 1 & 0 \\ 0 & -1 \end{pmatrix} \cdot \frac{1}{\sqrt{2}} \begin{pmatrix} 1 & -i \\ -i & 1 \end{pmatrix} \cdot \begin{pmatrix} \cos\frac{\theta}{2} & \sin\frac{\theta}{2} \\ \sin\frac{\theta}{2} & -\cos\frac{\theta}{2} \end{pmatrix} \cdot \frac{1}{\sqrt{2}} \begin{pmatrix} 1 & -i \\ -i & 1 \end{pmatrix} \\ &= e^{-i\frac{\theta}{2}} \begin{pmatrix} 1 & 0 \\ 0 & e^{i\theta} \end{pmatrix}, \end{aligned} \tag{S17}$$

where each term represents the standard Jones matrix of the corresponding wave plate. This operation imparts a phase $e^{i\theta}$ to each $|1\rangle$ component, resulting in the transformed GHZ state

$$|\mathrm{GHZ}_N(\theta)\rangle = \frac{1}{\sqrt{2}} \left(|0\rangle^{\otimes N} + e^{iN\theta} |1\rangle^{\otimes N} \right). \tag{S18}$$

We implement a near-optimal collective measurement $M = \sigma_x^{\otimes N}$ for system sizes $N = 2, \ldots, 6$ and experimentally determine the phase precision via error propagation:

$$(\Delta\theta)^2 = \frac{(\Delta M)^2}{|\partial_\theta \langle M \rangle|^2} = \frac{1 - \langle M \rangle^2}{|\partial_\theta \langle M \rangle|^2}.$$

The derivative $\partial_\theta \langle M \rangle$ is approximated using the five-point stencil method (*53*). For each N, we measure the average value of M at five phase settings around $\theta = 20^\circ$ with a step size of 4°.

B.7 Data Acquisition

We collected 1000 measurement shots for each of the 3^N Pauli bases, corresponding to a total of $3^N \times 1000$ measurement outcomes for an N-qubit state. Thus, the total numbers of measurement outcomes equal 9×1000, 27×1000, 81×1000, 243×1000, and 729×1000 for $N = 2, 3, 4, 5, 6$, respectively.

To reconstruct the density matrix via full quantum state tomography, we utilized the entire dataset containing all available measurement outcomes. In contrast, to evaluate the data efficiency of the Krylov shadow tomography protocol, we deliberately restricted the classical shadow estimators to a predefined subset of the total measurements. Depending on the specific analytical requirement, the size of this restricted subset spans from 10% to 80% of the full data.

To rigorously evaluate the statistical fluctuations and variance of the Krylov estimators under these restricted data regimes, we employed a formal nonparametric bootstrap procedure. For a given restricted dataset, we generated 100 independent bootstrap samples by randomly drawing measurements with replacement, maintaining the sample size identical to the specific restricted subset size. The quantum Fisher information values reported in the figures represent the mean of this bootstrapped ensemble, and the error bars correspond to the formal standard deviation evaluated across the 100 independent bootstrap realizations.

Furthermore, as quantum experimental systems inevitably experience phenomena such as environmental drift, the generated data is not strictly independent and identically distributed (i.i.d.). The underlying data structure often exhibits non-stationary and non-i.i.d. properties. While standard nonparametric bootstrap methods can theoretically accommodate such features, a more cautious algorithmic implementation, such as block bootstrapping, is required to rigorously capture the variance. To validate our statistical methodologies within a simplified and controlled regime, we conducted a comparative analysis using a 2-qubit system ($p = 0.4$), as illustrated in Fig. S7. For this evaluation, we utilized a subset of $9 \times 1000 \times 80\%$ samples and increased the number of independent bootstrap replications to 1000 to guarantee asymptotic validity. We compared the standard nonparametric approach (effectively a block size of 1) against block bootstrapping with block sizes (BS) of 50, 100, and 200. As shown in the figure, the estimated means and variances remain highly consistent across all block sizes, and the KST method ($B_1^{(\mathrm{Kry})}$) consistently maintains its operational advantage over the Taylor-expanded schemes. This consistency indicates that the 2-qubit system is relatively stable with minimal drift. However, for more complex and larger-scale setups, such as 6-qubit systems, drift-induced non-i.i.d. effects become more pronounced, making block bootstrapping a necessary and effective tool for accurately capturing the true statistical variance.

To estimate $(\Delta\theta)^2$, we employ the five-point stencil method based on experimentally mea-

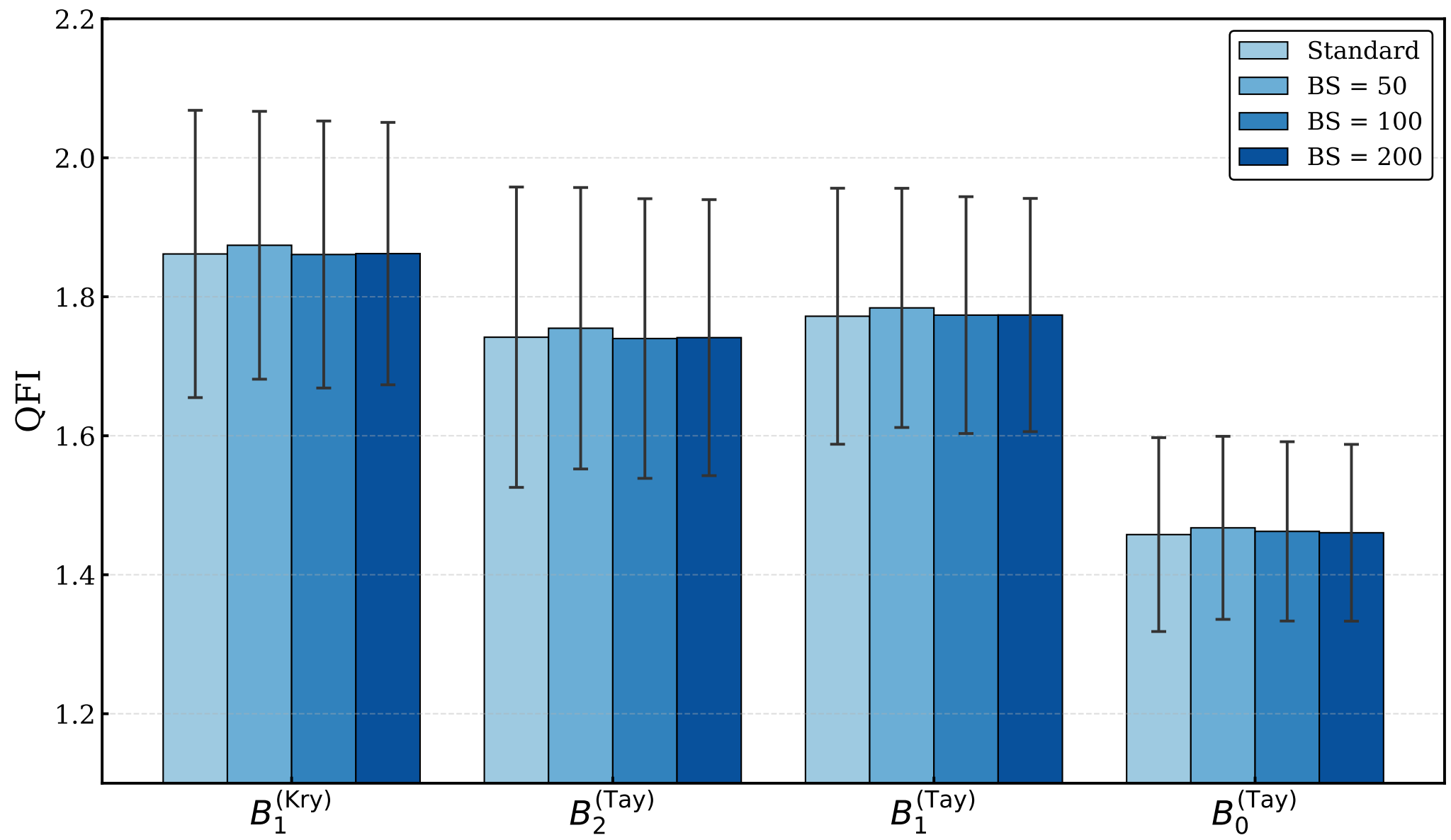

Figure S7: **Comparison of standard nonparametric bootstrap and block bootstrap methods.** The evaluation is performed on a 2-qubit system ($p = 0.4$) using a subset of $9 \times 1000 \times 80\%$ samples. The bar heights represent the mean estimated values for the Krylov-based estimator ($B_1^{(\mathrm{Kry})}$) and Taylor-expanded estimators ($B_2^{(\mathrm{Tay})}$, $B_1^{(\mathrm{Tay})}$, and $B_0^{(\mathrm{Tay})}$). The error bars indicate the standard deviation obtained from 1000 independent bootstrap replications, ensuring asymptotic validity. The color gradient distinguishes the standard nonparametric bootstrap (Standard, block size = 1) from block bootstraps with sizes (BS) of 50, 100, and 200. The highly consistent variance across different block sizes confirms the validity of the statistical estimation in this stable regime.

sured expectation values at different phase settings. For each measurement setting, the photon number determines the number of state copies that we use in the experiment. Since the two-photon source exhibits a much higher count rate than the four-photon source, we collect a smaller total number of copies for the four-photon experiments, even though we use longer integration times. Specifically, for the two-qubit states, we collected approximately 2.7×10^4 copies for each measurement configuration. For the three- to six-qubit states, due to the reduced count rate of the four-photon entanglement source, we used approximately 1.5×10^3 copies per configuration.

The error bars in Fig. 3 represent one standard deviation, which we obtain by assuming Poissonian counting statistics. Specifically, we generate 100 synthetic datasets by sampling from a Poisson distribution with the experimentally measured photon counts as the mean values. For each dataset, we re-evaluate $(\Delta\theta)^2$ and take the standard deviation of these values as the uncertainty. As a result, the two-qubit case exhibits smaller error bars compared to the three- to six-qubit cases. This difference mainly stems from the larger number of copies that we use in the measurements.

For the KST-based estimation, the data usage and error-bar estimation follow the same procedure that we describe for Fig. 2 of the main text.

C Estimation of QFI via KST

C.1 Mathematical Formulation of KST

We review an efficient protocol to estimate the Quantum Fisher Information via KST, $B_n^{(\mathrm{Kry})}$, by leveraging the classical shadow formalism. Let us recapitulate that $B_n^{(\mathrm{Kry})}$ can be expressed as follows (*41*):

$$B_n^{(\mathrm{Kry})} = b^T A^{-1} b, \tag{S19}$$

where A is an $n \times n$ matrix with its (i, j)-th entry given by T_{i+j-1} and b is an n-dimensional vector with its i-th entry given by T_{i-1}. Here, T_k is a polynomial of ρ, defined as

$$T_k := \mathrm{tr}\left(i[\rho, H]\mathcal{R}_\rho^k(i[\rho, H])\right). \tag{S20}$$

We arrive at the general expression of T_k:

$$T_k = \frac{1}{2^k}\sum_{l=0}^{k+2} \mu_l^{(k)}\,\mathrm{tr}\left(H\rho^l H\rho^{k-l+2}\right), \tag{S21}$$

where

$$\mu_l^{(k)} = \binom{k}{l} - 2\binom{k}{l-1} + \binom{k}{l-2}. \tag{S22}$$

Here, by convention, we set $\binom{k}{l} = 0$ when $k < l$ or when $l < 0$. Each observable T_k can thus be formulated as the expectation value over multiple copies of the state:

$$T_k = \mathrm{tr}\left[O^{(k+2)}\rho^{\otimes(k+2)}\right], \tag{S23}$$

where the operator $O^{(k+2)}$ is a specific linear combination of permutation operators acting on $k+2$ copies of the system:

$$O^{(k+2)} = \frac{1}{2^k}\sum_{l=0}^{k+2} \mu_l^{(k)}\,\Pi^{(k+2)}\left(H \otimes \mathbb{I}^{\otimes(l-1)} \otimes H \otimes \mathbb{I}^{\otimes(k-l+1)}\right). \tag{S24}$$

Here, $\Pi^{(k+2)}$ is a cyclic permutation operator defined by its action on a product state: $\Pi^{(k+2)}|\psi_1\rangle \otimes \cdots \otimes |\psi_{k+2}\rangle = |\psi_2\rangle \otimes \cdots \otimes |\psi_{k+2}\rangle \otimes |\psi_1\rangle$.

Therefore, to obtain $B_n^{(\mathrm{Kry})}$, we need to estimate all the T_k for $k = 0, \cdots, 2n-1$. The protocol requires estimating a series of multi-copy observables $\{T_k\}_{k=0}^{2n-1}$, which are then used to compute the estimate $\hat{B}_n^{(\mathrm{Kry})}$. Following the detailed analysis in Ref. (*41*), this MoM protocol can achieve a desired accuracy ϵ with failure probability δ for all $2n$ estimates simultaneously. This is guaranteed by choosing the number of groups K_1 and the number of snapshots per group $L = M/K_1$ as follows:

$$K_1 = 8\ln\frac{2n}{\delta}, \tag{S25}$$

and

$$L = \max_{1 \leq l \leq k+2n+1} \left[\frac{(4(k+2)(k+2))^2 \operatorname{tr}\left(\left[O_l^{(k+2)} \right]^2 \right)}{l!(k-l+2)!^2 \epsilon^2} \right]^{1/l} \frac{1}{2^l} \left(2^N + l - 1 \right), \tag{S26}$$

where the maximum is taken over all relevant indices k and l. The operator $O_l^{(k+2)}$ is derived from a symmetrized version of $O^{(k+2)}$:

$$O_l^{(k+2)} = \operatorname{tr}_{l+1,\ldots,k+2} \left[\overline{O}^{(k+2)} \left(\mathbb{I}^{\otimes l} \otimes \rho^{\otimes (k-l+2)} \right) \right], \tag{S27}$$

with $\overline{O}^{(k+2)} = \frac{1}{(k+2)!} \sum_\pi \pi^\dagger O^{(k+2)} \pi$, where π is a permutation operator over the $k+2$ copies.

C.2 Robust Estimation via Batch Shadows

As detailed in our recent work (*41*), this task can be accomplished by employing shadow tomography, which enables the estimation of polynomial functions of the density matrix ρ through U-statistics (*44*). The detailed procedure for computing the robust Krylov and Taylor bounds is summarized in Table S1.

To empirically estimate $\{T_k\}$, we generate M classical shadows of the state ρ. This involves repeatedly applying a random unitary from the single-qubit Clifford group, $U^{(m)} = \bigotimes_{j=1}^N u_j^{(m)}$, to the state ρ, followed by a measurement in the computational basis to obtain a bitstring $s^{(m)}$. Each measurement pair $(U^{(m)}, s^{(m)})$ yields a classical snapshot (or shadow) of the state:

$$\hat{\rho}^{(m)} = \bigotimes_{j=1}^N \left(3 u_j^{(m)\dagger} |s_j^{(m)}\rangle\langle s_j^{(m)}| u_j^{(m)} - \mathbb{I} \right). \tag{S28}$$

For a general multi-copy functional $X_c = \operatorname{Tr}(O^{(c)} \rho^{\otimes c})$, the standard U-statistic estimator calculates the observable by summing over all combinations of c distinct shadows from the M available samples:

$$\hat{X}_c = \frac{1}{c!} \binom{M}{c}^{-1} \sum_{m_1 \neq \ldots \neq m_c} \operatorname{Tr}\left[O^{(c)} \bigotimes_{i=1}^c \hat{\rho}^{(m_i)} \right]. \tag{S29}$$

Function RobustBoundsEstimator($S(\rho; M), K_1, K_2, H$)

1. Import $S(\rho; M) = \{\hat{\rho}^{(1)}, \ldots, \hat{\rho}^{(M)}\}$; // `Load classical shadows`
2. Split $S(\rho; M)$ into $K_{\text{total}} = K_1 \times K_2$ equally sized subsets and compute batch shadows

$$\tilde{\rho}^{(b)} = \frac{1}{\lfloor M/K_{\text{total}} \rfloor} \sum_{j \in \text{Subset}_b} \hat{\rho}^{(j)}, \quad b = 1, \ldots, K_{\text{total}};$$

// `Construct batch shadows`

3. Partition the K_{total} batch shadows into K_1 independent groups $G_1, \ldots, G_{K_1}$ with each group containing K_2 batch shadows;
4. **For** each c-copy target functional $X^{(c)}$ and for each group $g \in \{1, \ldots, K_1\}$ **do**

$$\hat{X}_g \leftarrow \frac{1}{|P_c|} \sum_{p \in P_c} \mathrm{tr}\left(O_X^{(c)} \bigotimes_{j=1}^{c} \tilde{\rho}_{p_j}^{(g)} \right);$$

// `Compute U-statistics over permutations` P_c `within group` G_g

5. Extract the robust global estimate via the median across all groups

$$\hat{X}_{\text{MoM}} \leftarrow \text{median}(\hat{X}_1, \ldots, \hat{X}_{K_1});$$

// `Apply Median of Means to suppress extreme outliers`

6. Execute Steps 4 and 5 to robustly estimate Krylov components $\hat{T}_0, \hat{T}_1, \hat{T}_2, \hat{T}_3$;
7. Compute Krylov bounds using the estimated components

$$\hat{B}_1^{(\text{Kry})} \leftarrow \frac{\hat{T}_0^2}{\hat{T}_1}, \quad A \leftarrow \begin{bmatrix} \hat{T}_1 & \hat{T}_2 \\ \hat{T}_2 & \hat{T}_3 \end{bmatrix}, \quad b \leftarrow \begin{bmatrix} \hat{T}_0 \\ \hat{T}_1 \end{bmatrix}, \quad \hat{B}_2^{(\text{Kry})} \leftarrow b^T A^{-1} b;$$

8. Execute Steps 4 and 5 to estimate Taylor trace combinations and compute bounds $\hat{B}_0^{(\text{Tay})}, \hat{B}_1^{(\text{Tay})}, \hat{B}_2^{(\text{Tay})}$;
9. **Return** Krylov bounds $\{\hat{B}_1^{(\text{Kry})}, \hat{B}_2^{(\text{Kry})}\}$ and Taylor bounds $\{\hat{B}_0^{(\text{Tay})}, \hat{B}_1^{(\text{Tay})}, \hat{B}_2^{(\text{Tay})}\}$.

Table S1: Procedure for robust estimation of Krylov and Taylor bounds using Batch Shadows and Median of Means

However, this standard approach is computationally demanding, since iterating through all possible permutations of c different shadows leads to a classical post-processing cost that scales as $\mathcal{O}(M^c)$.

To mitigate this issue as well as the effects of statistical outliers, we employ the batch shadow formalism (*40, 58*) coupled with a median-of-means (MoM) estimator. The core idea is to partition the M shadows into $K_{\text{total}} = K_1 \times K_2$ equally sized subsets, and average the snapshots within each subset to create B_{total} batch shadows. Specifically, the b-th batch shadow is constructed as:

$$\tilde{\rho}^{(b)} = \frac{K_{\text{total}}}{M} \sum_{m \in \text{Subset}_b} \hat{\rho}^{(m)}. \tag{S30}$$

Each batch shadow remains an unbiased estimator of the underlying quantum state. These K_{total} batch shadows are then partitioned into K_1 independent groups, with each group G_g containing K_2 batch shadows. Within each group $g \in \{1, \ldots, K_1\}$, we construct an unbiased U-statistic estimate $\hat{X}_g$ for the target functional. Finally, the robust global estimate is extracted via the median across all K_1 groups:

$$\hat{X}_{\text{MoM}} = \text{median}\left\{\hat{X}_1, \hat{X}_2, \ldots, \hat{X}_{K_1}\right\}. \tag{S31}$$

By properly choosing the batch parameters, the time complexity drops drastically from $\mathcal{O}(M^c)$ to $\mathcal{O}(M)$, while preserving the precision of the estimator. The detailed step-by-step classical post-processing procedure for KST is summarized in Table S1.

C.3 Statistical Distributions of T_k

To mitigate the influence of statistical outliers and ensure numerical stability, the median values of the raw sample distributions were employed for the baseline calculations in both the 4-qubit (Fig. S8) and 6-qubit (Fig. S9) scenarios. The first-order bound, $B_1^{(\text{Kry})}$, was computed using the relation $B_1^{(\text{Kry})} = T_0^2/T_1$, yielding stable values of approximately 7.476 for the 4-qubit case and

16.375 for the 6-qubit case. Furthermore, to capture the multidimensional correlations within the datasets, the second-order bound $B_2^{(\mathrm{Kry})}$ was extracted via a relation involving a matrix inversion. Specifically, it was evaluated as $B_2^{(\mathrm{Kry})} = b^T A^{-1} b$, where $b = [T_0, T_1]^T$, A represents the symmetric matrix constructed from $[[T_1, T_2], [T_2, T_3]]$, and A^+ denotes the Moore-Penrose pseudoinverse. This evaluation returned $B_2^{(\mathrm{Kry})} \approx 7.514$ and 18.376 for the 4-qubit and 6-qubit configurations, respectively, demonstrating a high degree of consistency with theoretical expectations and confirming the structural integrity of the extracted signals across different system sizes.

C.4 Evaluation of Higher-Order Krylov Bounds

However, given that the measurement costs and classical post-processing complexities inherent in shadow tomography scale exponentially with the Krylov order, identifying a practical truncation is paramount for near-term applications (*42*). This necessitates a careful examination of the trade-off between the increased theoretical tightness offered by higher-order bounds and the escalating experimental resource demands.

Motivated by these profound theoretical insights, our present work focuses primarily on the efficacy of the second-order Krylov estimator, $\hat{B}_2^{(\mathrm{Kry})}$, evaluated on our experimentally prepared multi-qubit states. Specifically, while the first-order bound relies on a straightforward ratio, $\hat{B}_1^{(\mathrm{Kry})} = \hat{T}_0^2/\hat{T}_1$, the second-order estimator incorporates deeper multidimensional correlations. It is constructed by defining a correlation matrix

$$A = \begin{bmatrix} \hat{T}_1 & \hat{T}_2 \\ \hat{T}_2 & \hat{T}_3 \end{bmatrix}, b = [\hat{T}_0, \hat{T}_1]^T$$

from the estimated Krylov components, and subsequently evaluating the quadratic form $\hat{B}_2^{(\mathrm{Kry})} = b^T A^{-1} b$. By leveraging this algebraic inversion, we demonstrate that the second-order KST yields a tighter lower bound than its first-order counterpart and conventional polynomial meth-

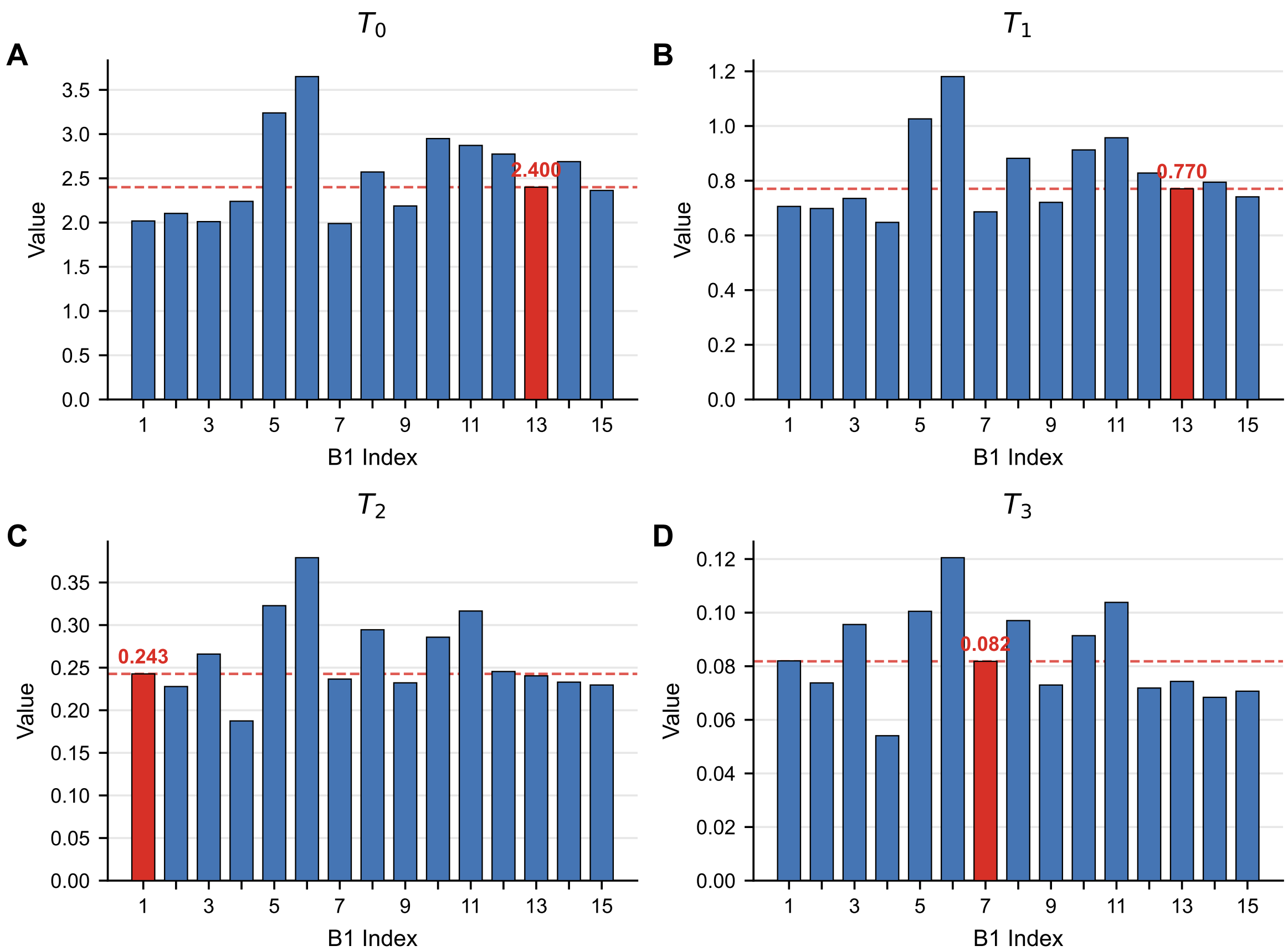

Figure S8: **Statistical distributions of the intermediate estimators for a noisy 4-qubit GHZ state.** (A to D) Raw sample distributions of the extracted variables T_0 (A), T_1 (B), T_2 (C), and T_3 (D), evaluated across 15 independent measurement batches. The data correspond to a state subjected to isotropic white noise with a mixing probability of $p = 0.4$. In each panel, the highlighted red bar and corresponding horizontal dashed line indicate the median value of the distribution, which is utilized as a robust input for calculating the global estimators $B_1^{(\mathrm{Kry})}$ and $B_2^{(\mathrm{Kry})}$.

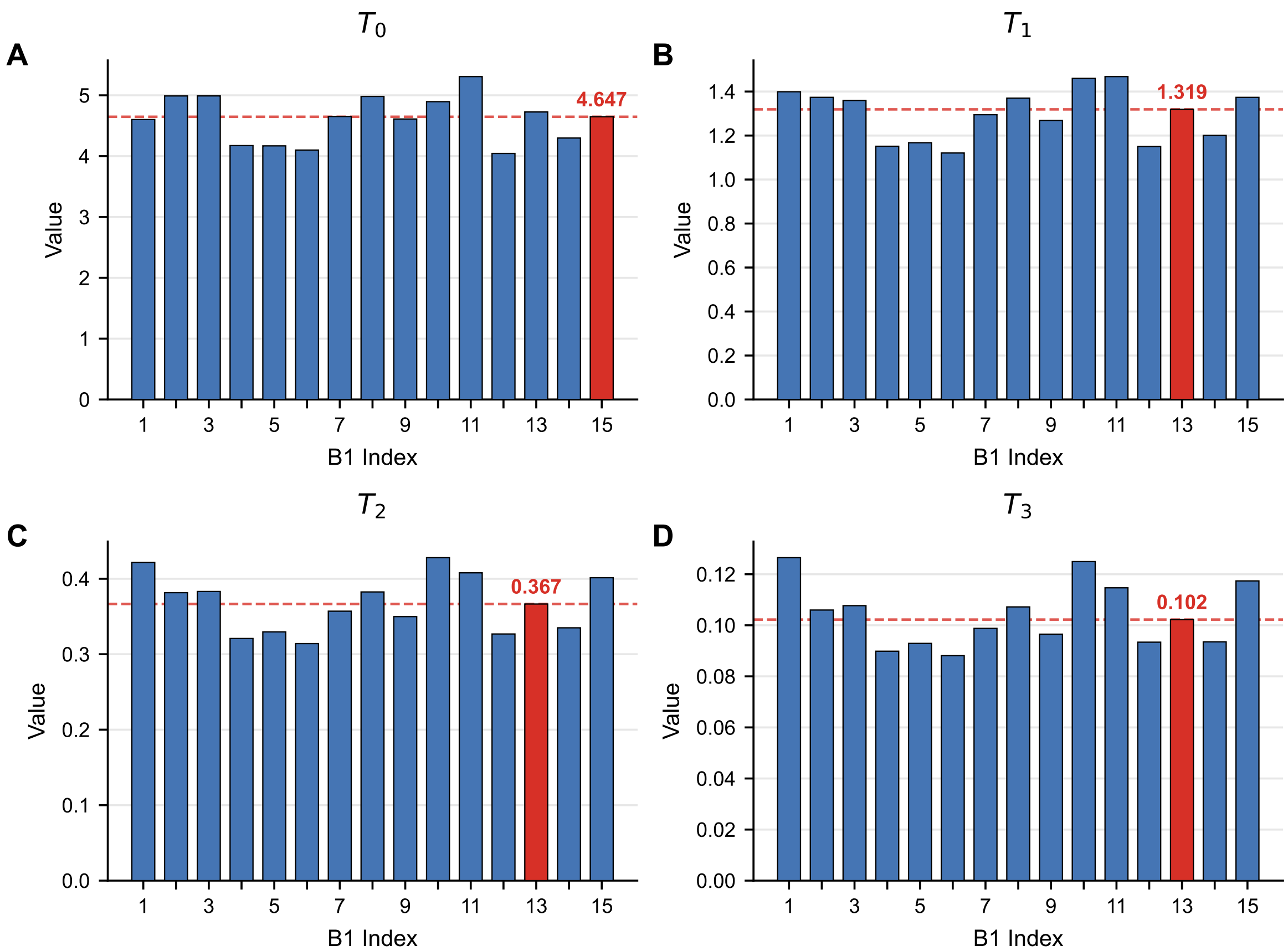

Figure S9: **Statistical distributions of the intermediate estimators for a noisy 6-qubit GHZ state.** (A to D) Raw sample distributions of the extracted variables T_0 (A), T_1 (B), T_2 (C), and T_3 (D), evaluated across 15 independent measurement batches. The data correspond to a state subjected to isotropic white noise with a mixing probability of $p = 0.4$. In each panel, the highlighted red bar and corresponding horizontal dashed line indicate the median value of the distribution, which is utilized as a robust input for calculating the global estimators $B_1^{(\mathrm{Kry})}$ and $B_2^{(\mathrm{Kry})}$.

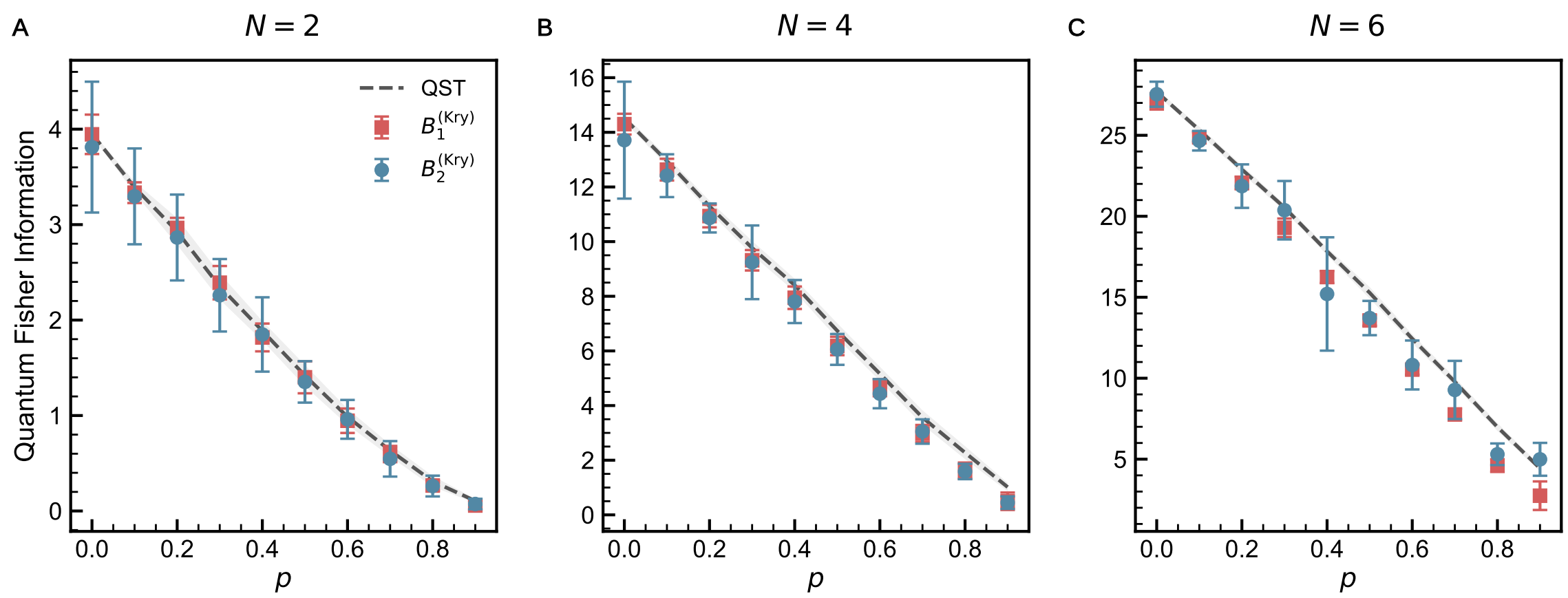

Figure S10: **Experimental estimation of QFI via second-order KST.** Panels A, B, and C display the estimated QFI as a function of the noise probability parameter p for system sizes $N = 2, 4$, and 6, respectively. The dashed grey line represents the exact QFI evaluated from QST, serving as the experimental baseline. The red squares and blue circles denote the experimental results obtained using the first-order ($\hat{B}_1^{(\mathrm{Kry})}$) and second-order ($\hat{B}_2^{(\mathrm{Kry})}$) Krylov estimators, respectively.

ods, while maintaining essential robustness against finite-sampling statistical noise in practical experimental regimes.

As illustrated in the experimental results for varying system sizes N shown in Fig. S10, both estimators track the baseline provided by quantum state tomography across the noise parameter regime p. Theoretically, for a GHZ state subjected to global white noise, the first-order Krylov bound $\hat{B}_1^{(\mathrm{Kry})}$ is sufficient to yield the exact QFI. However, experimental preparations contain uncharacterized noise channels beyond simple white noise. To assess the estimation reliability under these practical conditions, we evaluated the second-order bound $\hat{B}_2^{(\mathrm{Kry})}$ for the $N = 2$, 4, and 6 qubit systems. Under identical data volumes, the mean values yielded by $\hat{B}_2^{(\mathrm{Kry})}$ are consistent with $\hat{B}_1^{(\mathrm{Kry})}$. This agreement indicates the robustness of the first-order estimator and shows the convergence of the Krylov sequence for the prepared experimental states. Additionally, the experimental data show a larger standard deviation associated with the second-order estimator. This statistical behavior is expected within the shadow tomogra-

phy framework. The algebraic construction of $\hat{B}_2^{(\mathrm{Kry})}$ requires the evaluation of higher-order correlation moments, namely $\hat{T}_2$ and $\hat{T}_3$, and performing matrix inversions. Extracting these higher-order terms from the same finite ensemble of randomized measurements increases the sampling variance. While the measurement overhead for these higher-order bounds is theoretically manageable for ideal independent and identically distributed quantum states, our realistic experimental data inevitably deviates from perfect independent and identically distributed conditions due to slight system drifts. Consequently, evaluating these higher-order moments via shadows introduces significant statistical fluctuations, which are further amplified during the matrix inversion process. Furthermore, as the system size increases, particularly for the 6-qubit system, the inevitable reduction in quantum state preparation fidelity further degrades the signal-to-noise ratio, making the extraction of higher-order signals inherently highly unstable. From a computational perspective, under our specific batch shadows scheme, the classical computational effort required to process the massive number of higher-order shadow combinations for larger system sizes becomes practically prohibitive. Therefore, the larger error bars reflect the trade-off between the theoretical depth of the estimator and its statistical precision given a fixed measurement budget, establishing the first-order approximation as the most robust and practical balance for realistic experimental settings.

C.5 Impact of Sampling Size on Estimation Accuracy

To investigate the impact of sampling size on estimation accuracy, we evaluate the performance of different estimation strategies across various sampling ratios generated via bootstrap resampling. By sampling with replacement from an initial 80% data subset to construct ratios ranging from 10% to 80%, we present the estimated values for a 4-qubit system in panel a and a 6-qubit system in panel b of Fig. S11. The Krylov subspace technique yields markedly different results compared to the Taylor expansion methods. Specifically, the first-order and second-order

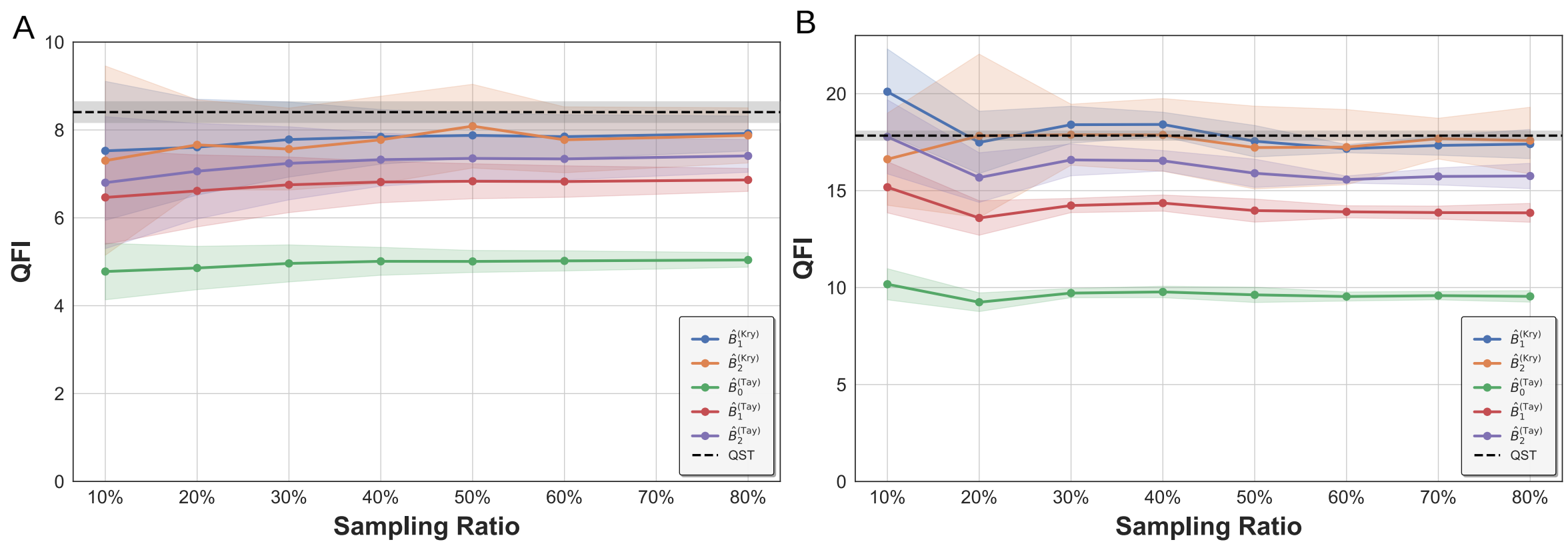

Figure S11: **Estimated QFI as a function of sampling ratio for noisy 4-qubit and 6-qubit systems ($p = 0.4$).** (A and B) Comparison of the Krylov Subspace Technique estimators ($\hat{B}_1^{(\mathrm{Kry})}$, $\hat{B}_2^{(\mathrm{Kry})}$) against Taylor expansion methods ($\hat{B}_0^{(\mathrm{Tay})}$, $\hat{B}_1^{(\mathrm{Tay})}$, $\hat{B}_2^{(\mathrm{Tay})}$) for 4-qubit (A) and 6-qubit (B) systems, evaluated using data volumes ranging from 10% to 80% of the total measurements. Solid lines represent the mean estimated values, while the corresponding shaded regions indicate ± 1 standard deviation across independent trials. The horizontal black dashed lines mark the exact target values established via quantum state tomography (8.4061 ± 0.2364 for the 4-qubit system and 17.8381 ± 0.2371 for the 6-qubit system).

Krylov estimators $\hat{B}_1^{(\mathrm{Kry})}$ and $\hat{B}_2^{(\mathrm{Kry})}$ perform better than the Taylor approximations $\hat{B}_0^{(\mathrm{Tay})}$, $\hat{B}_1^{(\mathrm{Tay})}$, and $\hat{B}_2^{(\mathrm{Tay})}$ even when only a small fraction of the data is utilized. The Taylor series estimators show a systematic underestimation and do not converge to the target standard value as the data amount increases. In contrast, the Krylov estimators converge to the theoretical baseline across both system sizes. Furthermore, because the second-order Krylov estimator $\hat{B}_2^{(\mathrm{Kry})}$ requires estimating more parameters, it introduces higher statistical variance and is more sensitive to sampling noise. As a result, the first-order Krylov estimator $\hat{B}_1^{(\mathrm{Kry})}$ shows a more stable convergence behavior.

C.6 Estimation of QFI for N = 2, 3, 5 Qubits

In this section, we present the comprehensive experimental data for GHZ states with $N = 2, 3, 5$ qubits, as shown in Fig. S12. These results corroborate the conclusions presented in the main

text for the $N = 4, 6$ case, demonstrating the consistent superiority of the KST protocol across all system sizes studied.

D Quantum state characterization

D.1 QST Method Based on Convex Optimization

In contrast, the QST-based estimation follows a different statistical treatment. We use the full dataset consisting of $3^N \times 1000$ measurement outcomes to compute their respective expectation values. These values then serve as inputs for the reconstruction algorithm described below. To account for finite-shot noise, error bars for the QST results are determined by modeling the measurement frequencies as a Poisson distribution. Each error bar is derived from 300 independent Monte Carlo simulations of the measurement statistics. We employ a widely used convex optimization approach (*63–66*) for QST which is given by

$$\min_{\mathbf{x}_\rho} \|\mathcal{A}\mathbf{x}_\rho - \mathcal{B}\|_{l_2} + \lambda \|\mathbf{x}_\rho\|_{l_1} \quad \text{s.t.} \quad \rho \geq 0, \text{Tr}(\rho) = 1 \tag{S32}$$

where $\|\cdot\|_{l_2}$ and $\|\cdot\|_{l_1}$ denote the L_2 and L_1 norm, respectively. In this formulation, $\mathcal{B}$ is a vector constructed from the experimental measurement outcomes, corresponding to the expectation values of the operators in a tomographically complete set, which in our case consists of the 4^N Pauli operators. The expectation values for all 4^N operators, including those involving the identity matrix, can be directly derived from the experimental data collected using the 3^N Pauli measurement bases. The sensing matrix $\mathcal{A}$ is defined by the measurement scheme, with each element given by $\mathcal{A}_{ij} = \text{Tr}(M_i P_j)$, where $\{M_i\}$ is the set of measurement operators and $\{P_j\}$ is the operator basis used to expand the density matrix. This matrix maps the vectorized density matrix $\mathbf{x}_\rho$ (the coefficients of ρ in the basis $\{P_j\}$) to the ideal measurement outcomes. To ensure physical validity, the reconstructed density matrix is strictly defined as a Hermitian variable within the optimization space. The L_2 norm is the standard Euclidean norm of a vector, while

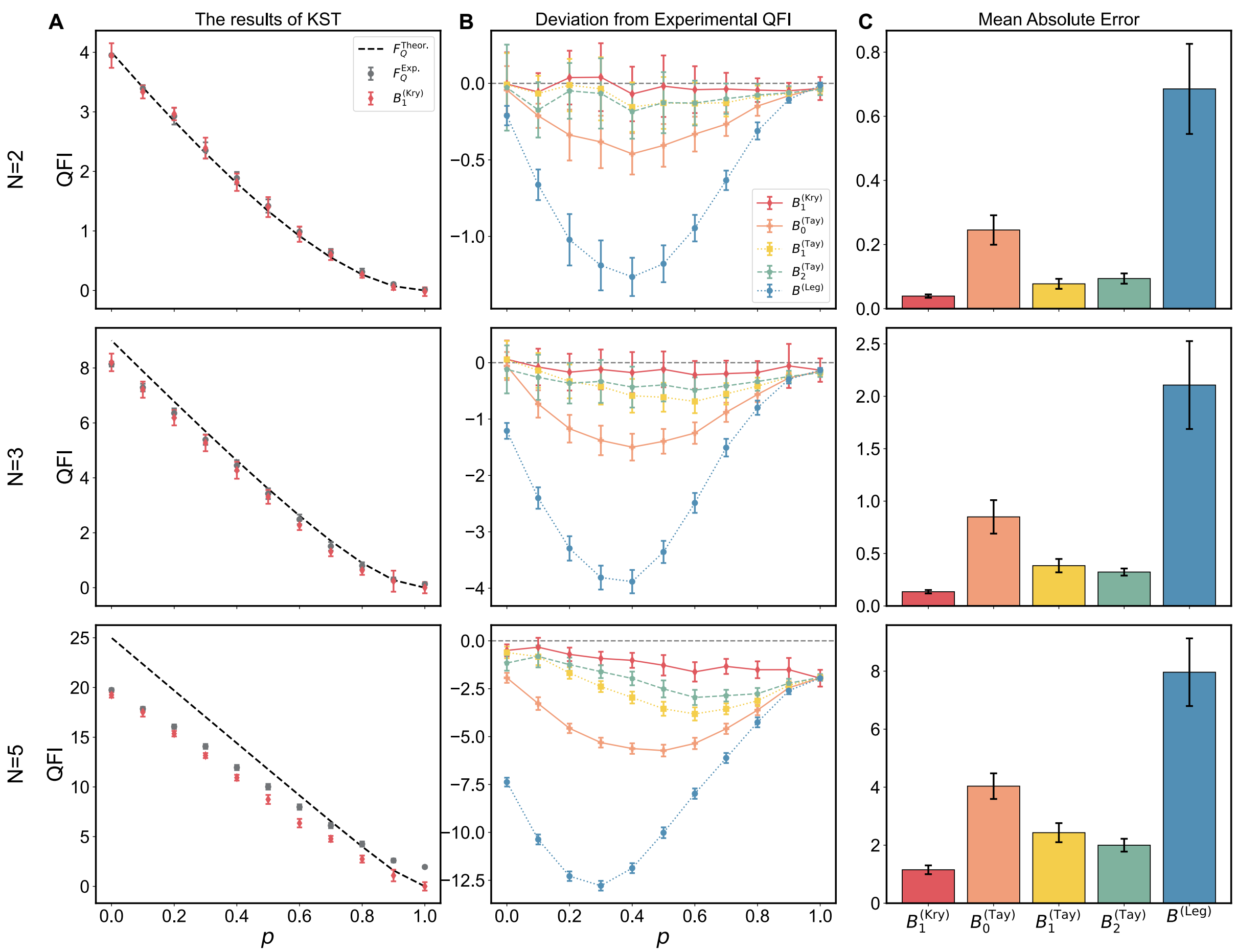

Figure S12: **Evaluation of QFI estimation protocols for 2-, 3-, and 5-qubit GHZ states under varying white noise levels.** Results are displayed as a function of the noise parameter p, partitioned into rows for $N = 2$ (top row), $N = 3$ (middle row), and $N = 5$ (bottom row) systems. (A) Comparison of the QFI estimated via the KST protocol ($B_1^{(\text{Kry})}$, red diamonds) against the theoretical baseline ($F_Q^{\text{Theor.}}$, dashed black line) and experimental quantum state tomography results ($F_Q^{\text{Exp.}}$, grey curve). (B) Deviation of each evaluated estimation protocol from the experimental tomography baseline ($F_Q^{\text{Exp.}}$). (C) Statistical performance comparison showing the mean absolute error (MAE) for each protocol, averaged over all p values. Evaluated protocols include the KST estimator ($B_1^{(\text{Kry})}$, red diamonds), Taylor expansion-based methods $B_0^{(\text{Tay})}$ (orange), $B_1^{(\text{Tay})}$ (yellow), $B_2^{(\text{Tay})}$ (green), and Legendre (blue) protocols. Error bars represent standard deviations derived from 300 iterations of Poissonian resampling for experimental data or 100 rounds of random sampling for classical-shadow-based estimators.

the L_1 norm is the sum of the absolute values of all components. The scalar quantity λ controls the sparsity of the solution vector $\mathbf{x}_\rho$, which in this case is a reconstructed density matrix. For all system sizes evaluated in our experiment, this regularization parameter was uniformly set to a constant value of $\lambda = 1$. This approach is commonly solved using standard convex optimization packages such as `CVXPY` (*67*), where we specifically employed the SCS solver to compute the optimum. It is worth noting that for tomography on even larger quantum systems, a variety of other highly efficient protocols are also available (*68*).

D.2 The Tomographic Results of the Experimentally Prepared Quantum States

We present the quantum state tomography results for the experimentally prepared states referenced in the main text. The reconstructed density matrices are shown in Figs. S14–S18. In each figure, the subfigures (A) and (B) correspond to ρ_N^0 and ρ_N^1, respectively. Real and imaginary parts of the density matrices are presented. The fidelities $F(\rho_N(p), \sigma_N(p)) = \mathrm{Tr}\big(\sqrt{\sqrt{\rho}\,\sigma\,\sqrt{\rho}}\big)$ between the experimental states $\rho_N(p)$ and their respective ideal target states $\sigma_N(p)$ are summarized in Table S2.

To provide a clearer overview of the fidelity behavior, we plot the state fidelity as a function of the noise parameter p for $N = 2$ to 6 qubits in Fig. S13. As shown, the overall fidelity decreases with increasing system size, reflecting the accumulation of experimental imperfections in larger systems. Moreover, distinct trends are observed for different qubit numbers, which we analyze below.

In our experiment, the deviations from the ideal generalized Werner state arise from several well-identified noise mechanisms.

First, the SPDC sources introduce higher-order multi-photon emissions. Second, for the four-photon entanglement source, imperfect multi-photon interference at the spatial PBS further

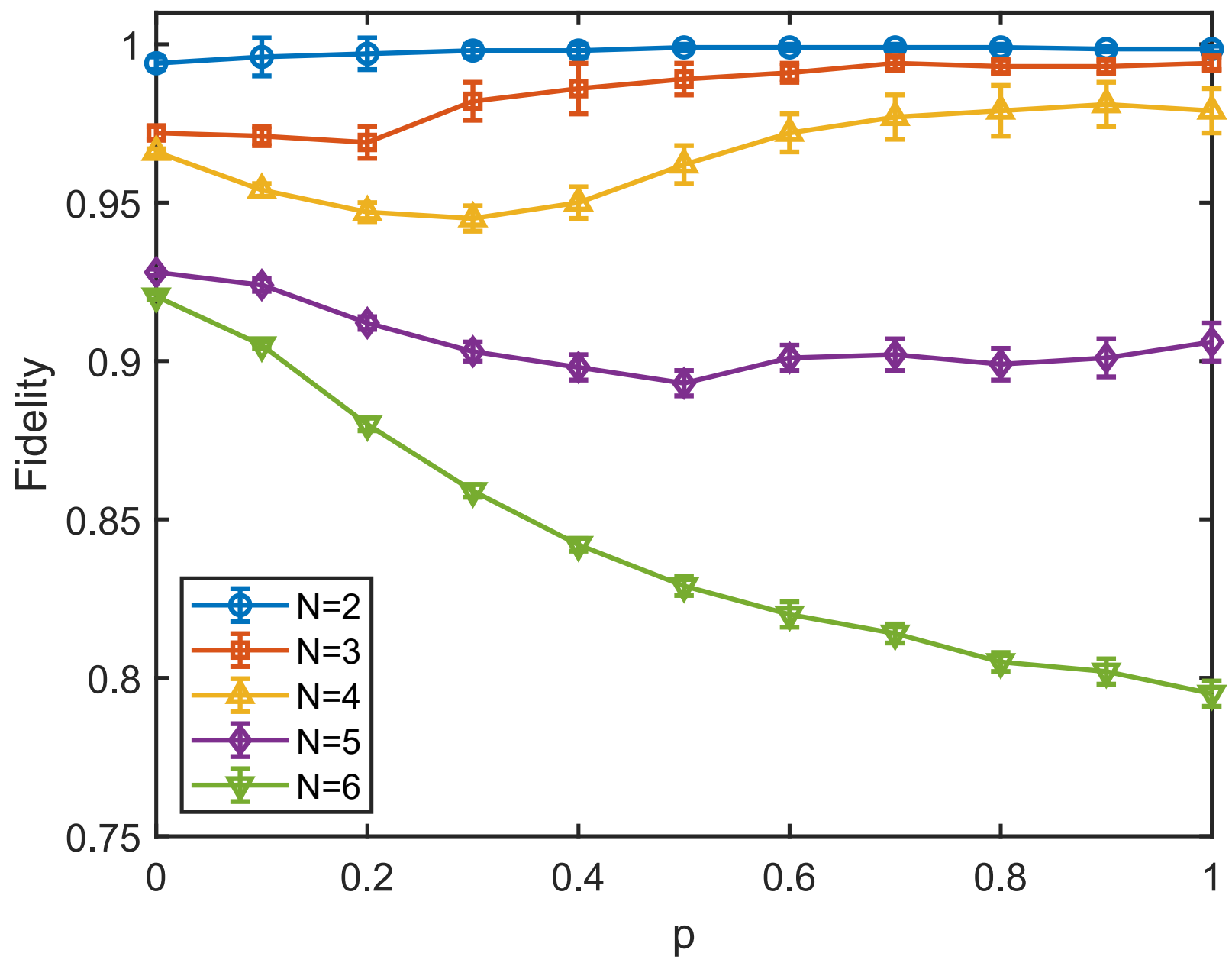

Figure S13: **State fidelity as a function of the noise parameter p for $N = 2$ to 6 qubits.** The plotted data illustrate the fidelity of the experimentally prepared states across varying white noise levels for different system sizes. Error bars represent one standard deviation.

degrades the state fidelity. Third, when encoding additional qubits in the path degree of freedom using BDs, extra noise is introduced due to path-mode mismatch and interferometric instability. Fourth, the experimentally realized states deviate from the ideal maximally mixed state, leading to imperfect depolarizing noise.

For the 2–4 qubit states, the photon-pair production rate of the entanglement source is set to 0.007. In this regime, the two-qubit states are mainly affected by higher-order emissions and imperfect white noise, resulting in the highest fidelity; the three- and four-qubit states are additionally affected by imperfect multi-photon interference at the PBS.

For the 5- and 6-qubit states, the number of measurement settings scales as 3^N, leading to a significant increase in data acquisition time. To reduce the measurement time, we increase the count rate by raising the photon-pair production rate of the entanglement source to 0.04,

which in turn enhances the higher-order emission noise. In addition, the use of path encoding introduces further noise from the BD-based interferometric structure.

Moreover, the effect of imperfect maximally mixed state becomes more pronounced for larger systems. Specifically, the diagonal elements of the maximally mixed state scale as $1/2^N$ (e.g., $1/32$ for 5 qubits and $1/64$ for 6 qubits), making the states more sensitive to deviations from the ideal depolarizing model compared to the two-qubit case $(1/4)$. As a result, the 5- and 6-qubit states are affected simultaneously by all four noise sources, with enhanced contributions from higher-order emissions and imperfect white noise, leading to a further reduction in fidelity.

For the $n = 3$ and $n = 4$ cases, the increase of the fidelity as $p \to 1$ originates from the fact that, in this regime, the target state is increasingly dominated by the mixed component of the generalized Werner state, while the weight of the pure GHZ component is progressively reduced. For the three- and four-qubit states, the dominant experimental imperfection mainly affects the preparation of the GHZ-like pure component, in particular through imperfect multi-photon interference at the spatial PBS, which lowers the fidelity of the pure-state part. As p increases, the contribution of this imperfect pure component is suppressed by the factor $(1-p)$, so its negative effect on the total fidelity becomes weaker. As a result, the overall fidelity increases when $p \to 1$, as also observed in Fig. S13.

By contrast, for the $n = 5$ and $n = 6$ cases, the situation is more complicated. In addition to the imperfections affecting the pure component, these states also suffer from extra noise introduced by the path-degree-of-freedom encoding and a more pronounced sensitivity to deviations from the ideal depolarizing-noise model. Therefore, even in the large-p regime, the mixed component may no longer be as close to the ideal one as in the $n = 3, 4$ cases, and consequently the fidelity does not exhibit a similar increasing trend, which is consistent with the behavior observed in Fig. S13.

Overall, the combined analysis of the reconstructed density matrices and the fidelity trends in Fig. S13 demonstrates that the experimentally realized states systematically deviate from the ideal generalized Werner states due to well-understood physical noise mechanisms.

Table S2: Fidelities of the experimental states $\rho_N(p)$.

State	Fidelity	State	Fidelity	State	Fidelity	State	Fidelity
$\rho_2(0)$	0.994 ± 0.002	$\rho_2(0.1)$	0.996 ± 0.006	$\rho_2(0.2)$	0.997 ± 0.005	$\rho_2(0.3)$	0.998 ± 0.002
$\rho_2(0.4)$	0.998 ± 0.002	$\rho_2(0.5)$	0.999 ± 0.001	$\rho_2(0.6)$	0.999 ± 0.001	$\rho_2(0.7)$	0.999 ± 0.001
$\rho_2(0.8)$	0.999 ± 0.001	$\rho_2(0.9)$	0.9985 ± 0.0009	$\rho_2(1)$	0.9985 ± 0.0009		
$\rho_3(0)$	0.972 ± 0.002	$\rho_3(0.1)$	0.971 ± 0.003	$\rho_3(0.2)$	0.969 ± 0.005	$\rho_3(0.3)$	0.982 ± 0.006
$\rho_3(0.4)$	0.986 ± 0.008	$\rho_3(0.5)$	0.989 ± 0.005	$\rho_3(0.6)$	0.991 ± 0.003	$\rho_3(0.7)$	0.994 ± 0.002
$\rho_3(0.8)$	0.993 ± 0.002	$\rho_3(0.9)$	0.993 ± 0.002	$\rho_3(1)$	0.994 ± 0.002		
$\rho_4(0)$	0.966 ± 0.001	$\rho_4(0.1)$	0.954 ± 0.002	$\rho_4(0.2)$	0.947 ± 0.003	$\rho_4(0.3)$	0.945 ± 0.004
$\rho_4(0.4)$	0.950 ± 0.005	$\rho_4(0.5)$	0.962 ± 0.006	$\rho_4(0.6)$	0.972 ± 0.006	$\rho_4(0.7)$	0.977 ± 0.007
$\rho_4(0.8)$	0.979 ± 0.008	$\rho_4(0.9)$	0.981 ± 0.007	$\rho_4(1)$	0.979 ± 0.007		
$\rho_5(0)$	0.928 ± 0.001	$\rho_5(0.1)$	0.924 ± 0.002	$\rho_5(0.2)$	0.912 ± 0.002	$\rho_5(0.3)$	0.903 ± 0.003
$\rho_5(0.4)$	0.898 ± 0.004	$\rho_5(0.5)$	0.893 ± 0.004	$\rho_5(0.6)$	0.901 ± 0.004	$\rho_5(0.7)$	0.902 ± 0.005
$\rho_5(0.8)$	0.899 ± 0.005	$\rho_5(0.9)$	0.901 ± 0.006	$\rho_5(1)$	0.906 ± 0.006		
$\rho_6(0)$	0.9204 ± 0.0009	$\rho_6(0.1)$	0.905 ± 0.001	$\rho_6(0.2)$	0.880 ± 0.002	$\rho_6(0.3)$	0.859 ± 0.002
$\rho_6(0.4)$	0.842 ± 0.002	$\rho_6(0.5)$	0.829 ± 0.003	$\rho_6(0.6)$	0.820 ± 0.004	$\rho_6(0.7)$	0.814 ± 0.003
$\rho_6(0.8)$	0.805 ± 0.003	$\rho_6(0.9)$	0.802 ± 0.004	$\rho_6(1)$	0.795 ± 0.004		

E Weighted least-squares fitting of Figure 3

To quantify the linear relationship shown in Figure 3, we performed a weighted least-squares fit to the data points, using the measured uncertainties along the y-axis ($\Delta 1/(\Delta\theta)^2$) as weights. The fitting function is linear, $y = ax + b$, and the optimal parameters (a, b) are obtained by minimizing the weighted sum of squared residuals. The resulting slope and intercept are reported in the main text. Note that only the y-axis uncertainties are considered in this fit, while the x-axis uncertainties are neglected, because the y-axis uncertainties are much larger than the x-axis uncertainties and are strongly influenced by the photon count rates for two- and multi-photon states.

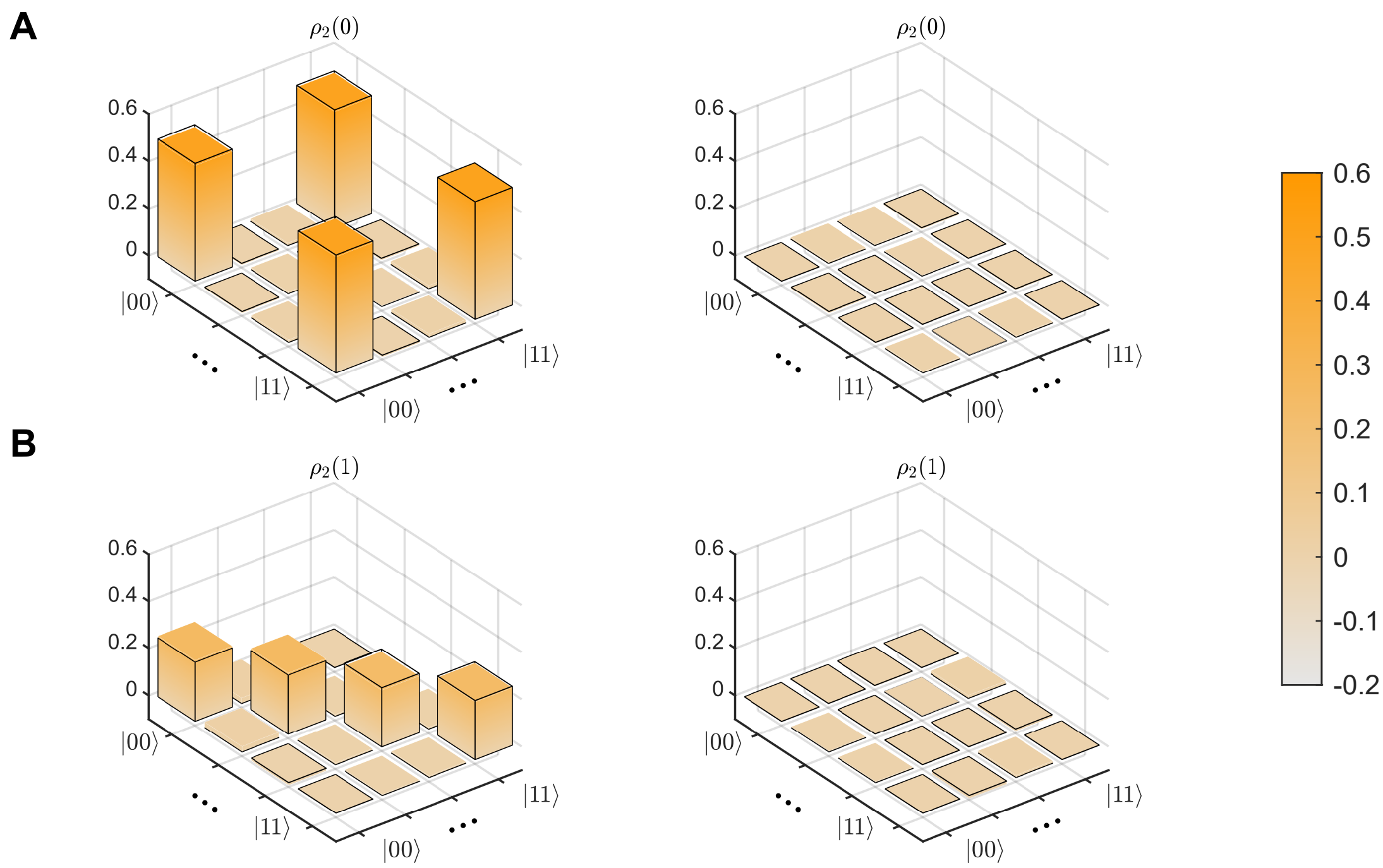

Figure S14: **Reconstructed density matrices for the 2-qubit state.** (A)$\rho_2(0)$. The real part is shown on the left, and the imaginary part on the right. (B)$\rho_2(1)$. The real part is shown on the left, and the imaginary part on the right.

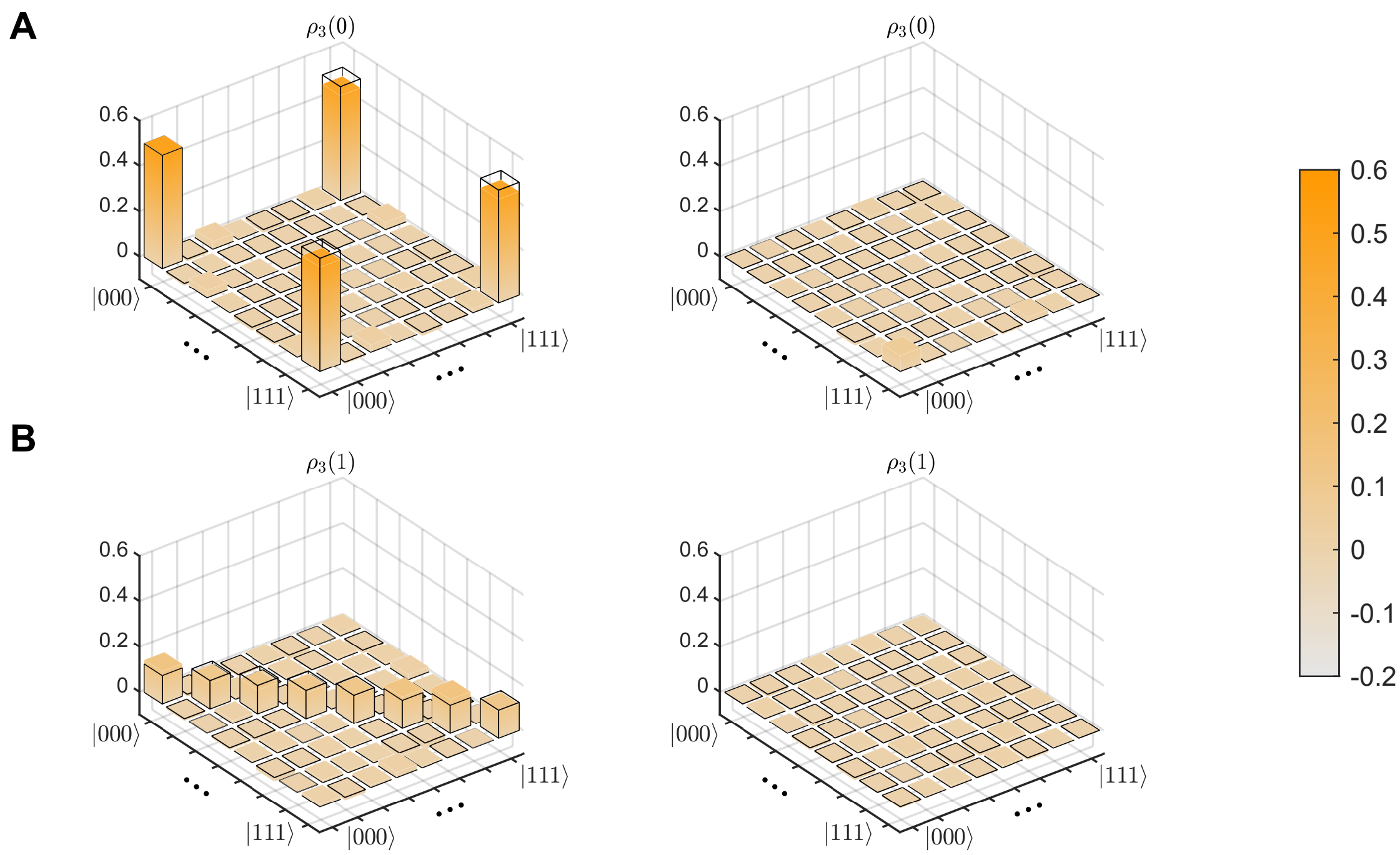

Figure S15: **Reconstructed density matrices for the 3-qubit state.** (A)$\rho_3(0)$. The real part is shown on the left, and the imaginary part on the right. (B)$\rho_3(1)$. The real part is shown on the left, and the imaginary part on the right.

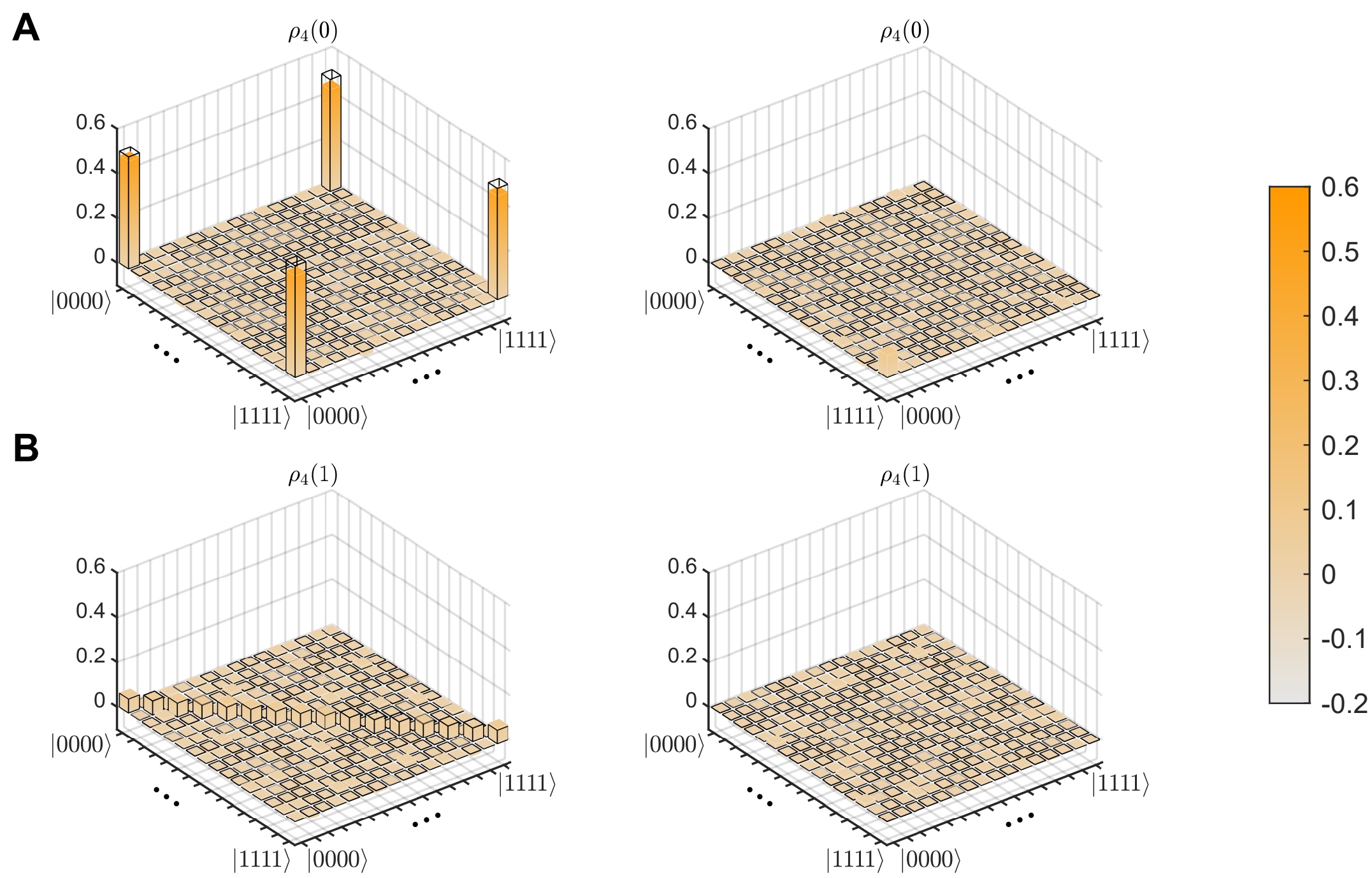

Figure S16: **Reconstructed density matrices for the 4-qubit state.** (A)$\rho_4(0)$. The real part is shown on the left, and the imaginary part on the right. (B)$\rho_4(1)$. The real part is shown on the left, and the imaginary part on the right.

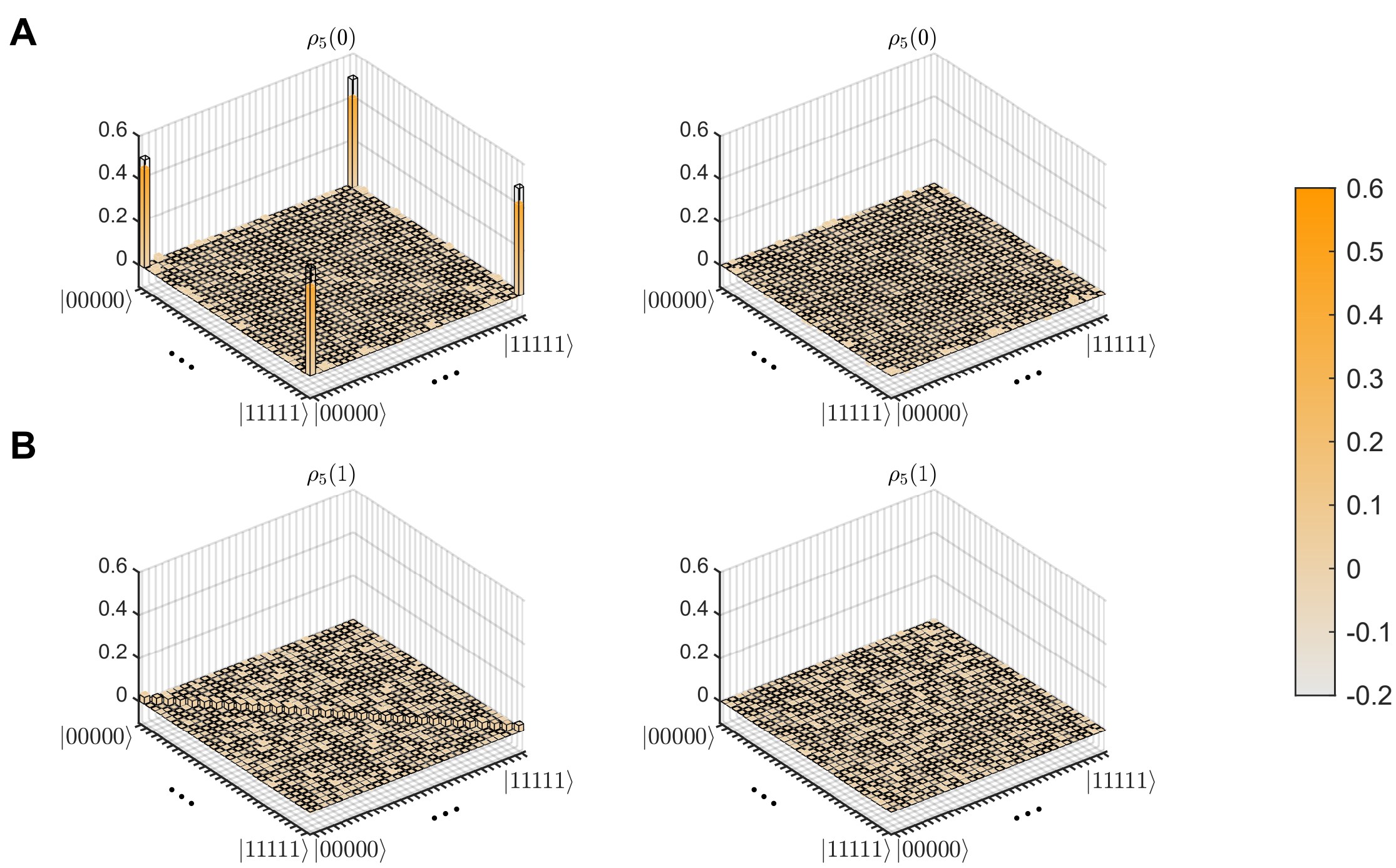

Figure S17: **Reconstructed density matrices for the 5-qubit state.** (A)$\rho_5(0)$. The real part is shown on the left, and the imaginary part on the right. (B)$\rho_5(1)$. The real part is shown on the left, and the imaginary part on the right.

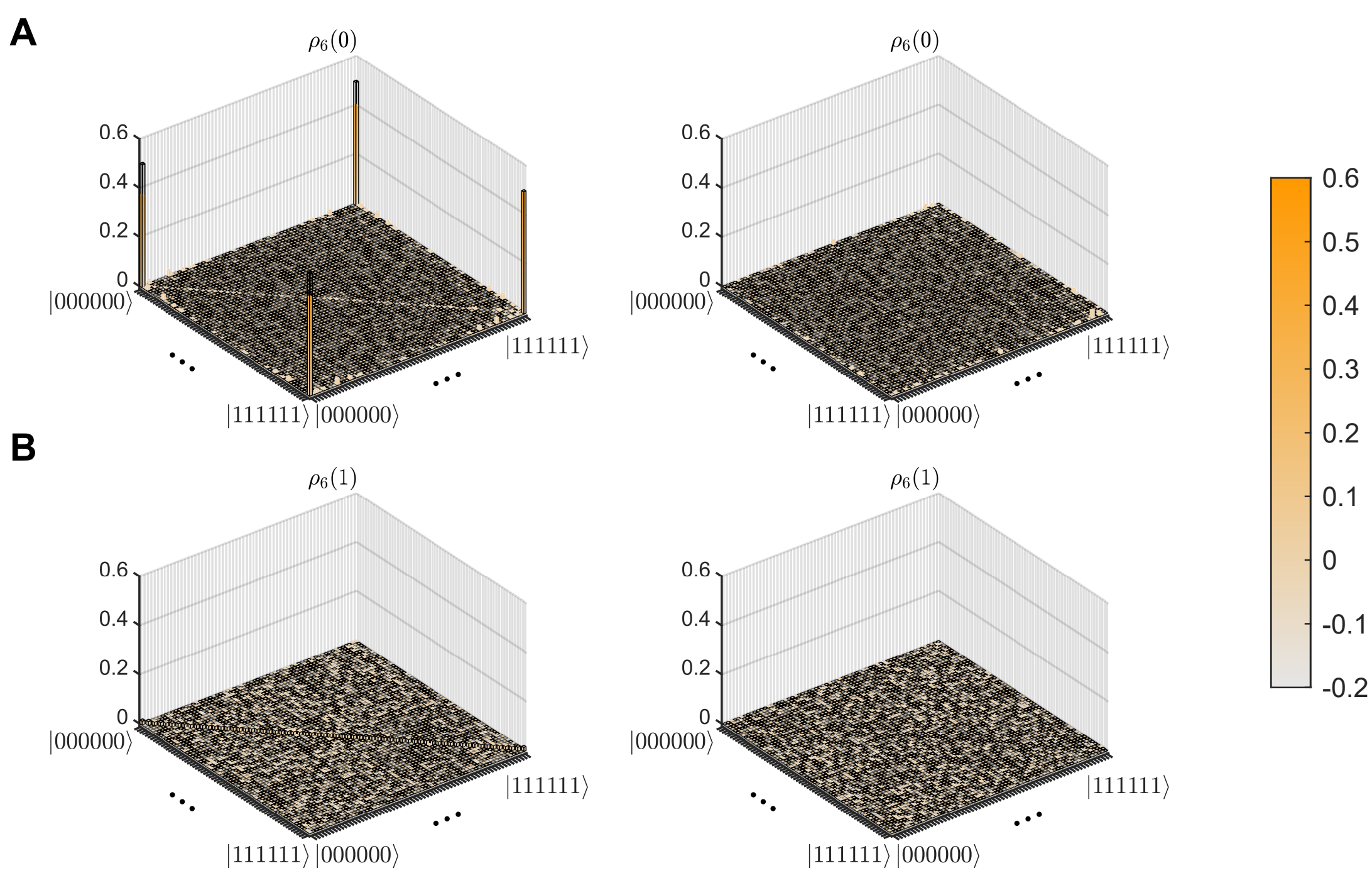

Figure S18: **Reconstructed density matrices for the 6-qubit state.** (A)$\rho_6(0)$. The real part is shown on the left, and the imaginary part on the right. (B)$\rho_6(1)$. The real part is shown on the left, and the imaginary part on the right.

REFERENCES

1. S. L. Braunstein, C. M. Caves, Statistical distance and the geometry of quantum states. *Phys. Rev. Lett.* **72**, 3439–3443 (1994).

2. S. L. Braunstein, C. M. Caves, G. Milburn, Generalized uncertainty relations: Theory, examples, and Lorentz invariance. *Ann. Phys.* **247**, 135–173 (1996).

3. V. Giovannetti, S. Lloyd, L. Maccone, Advances in quantum metrology. *Nat. Photonics* **5**, 222–229 (2011).

4. R. Demkowicz-Dobrzański, J. Kołodyński, M. Guţă, The elusive Heisenberg limit in quantum-enhanced metrology. *Nat. Commun.* **3**, 1063 (2012).

5. C. L. Degen, F. Reinhard, P. Cappellaro, Quantum sensing. *Rev. Mod. Phys.* **89**, 035002 (2017).

6. J. Liu, H. Yuan, X.-M. Lu, X. Wang, Quantum Fisher information matrix and multiparameter estimation. *J. Phys. A Math. Theor.* **53**, 023001 (2020).

7. M. G. A. Paris, Quantum estimation for quantum technology. *Int. J. Quantum Inf.* **07**, 125–137 (2009).

8. C. W. Helstrom, Minimum mean-squared error of estimates in quantum statistics. *Phys. Lett. A* **25**, 101–102 (1967).

9. G. Tóth, I. Apellaniz, Quantum metrology from a quantum information science perspective. *J. Phys. A Math. Theor.* **47**, 424006 (2014).

10. L. Pezzé, A. Smerzi, Entanglement, nonlinear dynamics, and the Heisenberg limit. *Phys. Rev. Lett.* **102**, 100401 (2009).

11. P. Hyllus, W. Laskowski, R. Krischek, C. Schwemmer, W. Wieczorek, H. Weinfurter, L. Pezzé, A. Smerzi, Fisher information and multiparticle entanglement. *Phys. Rev. A* **85**, 022321 (2012).

12. G. Tóth, Multipartite entanglement and high-precision metrology. *Phys. Rev. A* **85**, 022322 (2012).

13. Z. Ren, W. Li, A. Smerzi, M. Gessner, Metrological detection of multipartite entanglement from young diagrams. *Phys. Rev. Lett.* **126**, 080502 (2021).

14. Y. Yang, B. Yadin, Z.-P. Xu, Quantum-enhanced metrology with network states. *Phys. Rev. Lett.* **132**, 210801 (2024).

15. S. Imai, A. Smerzi, L. Pezzè, Metrological usefulness of entanglement and nonlinear Hamiltonians. *Phys. Rev. A* **111**, L020402 (2025).

16. S. Boixo, A. Monras, Operational interpretation for global multipartite entanglement. *Phys. Rev. Lett.* **100**, 100503 (2008).

17. G. Tóth, T. Vértesi, Quantum states with a positive partial transpose are useful for metrology. *Phys. Rev. Lett.* **120**, 020506 (2018).

18. G. Tóth, T. Vértesi, P. Horodecki, R. Horodecki, Activating hidden metrological usefulness. *Phys. Rev. Lett.* **125**, 020402 (2020).

19. K. F. Pál, G. Tóth, E. Bene, T. Vértesi, Bound entangled singlet-like states for quantum metrology. *Phys. Rev. Res.* **3**, 023101 (2021).

20. B. Yadin, M. Fadel, M. Gessner, Metrological complementarity reveals the Einstein-Podolsky-Rosen paradox. *Nat. Commun.* **12**, 2410 (2021).

21. K. C. Tan, V. Narasimhachar, B. Regula, Fisher information universally identifies quantum resources. *Phys. Rev. Lett.* **127**, 200402 (2021).

22. I. Gianani, V. Berardi, M. Barbieri, Witnessing quantum steering by means of the Fisher information. *Phys. Rev. A* **105**, 022421 (2022).

23. S. Szalay, G. Tóth, Alternatives of entanglement depth and metrological entanglement criteria. *Quantum* **9**, 1718 (2025).

24. L. Pezzè, Y. Li, W. Li, A. Smerzi, Witnessing entanglement without entanglement witness operators. *Proc. Natl. Acad. Sci. U.S.A.* **113**, 11459–11464 (2016).

25. A. Smerzi, Zeno dynamics, indistinguishability of state, and entanglement. *Phys. Rev. Lett.* **109**, 150410 (2012).

26. T.-L. Wang, L.-N. Wu, W. Yang, G.-R. Jin, N. Lambert, F. Nori, Quantum Fisher information as a signature of the superradiant quantum phase transition. *New J. Phys.* **16**, 063039 (2014).

27. K. Macieszczak, M. Guţă, I. Lesanovsky, J. P. Garrahan, Dynamical phase transitions as a resource for quantum enhanced metrology. *Phys. Rev. A* **93**, 022103 (2016).

28. T. Haug, M. S. Kim, Generalization of quantum machine learning models using quantum Fisher information metric. *Phys. Rev. Lett.* **133**, 050603 (2024).

29. X. Yu, X. Zhao, L. Li, X.-M. Hu, X. Duan, H. Yuan, C. Zhang, Toward Heisenberg scaling in non-Hermitian metrology at the quantum regime. *Sci. Adv.* **10**, eadk7616 (2024).

30. X. Zhao, X. Yu, L. Li, W. Zhou, C. Zhang, Factorization dynamics between quantum Fisher information and quantum coherence. *Sci. Adv.* **11**, eadv8132 (2025).

31. H. Strobel, W. Muessel, D. Linnemann, T. Zibold, D. B. Hume, L. Pezzè, A. Smerzi, M. K. Oberthaler, Fisher information and entanglement of non-Gaussian spin states. *Science* **345**, 424–427 (2014).

32. F. Fröwis, P. Sekatski, W. Dür, Detecting large quantum Fisher information with finite measurement precision. *Phys. Rev. Lett.* **116**, 090801 (2016).

33. M. Yu, Y. Liu, P. Yang, M. Gong, Q. Cao, S. Zhang, H. Liu, M. Heyl, T. Ozawa, N. Goldman, J. Cai, Quantum Fisher information measurement and verification of the quantum Cramér–Rao bound in a solid-state qubit. *npj Quantum Inf.* **8**, 56 (2022).

34. Á. Rivas, A. Luis, Precision quantum metrology and nonclassicality in linear and nonlinear detection schemes. *Phys. Rev. Lett.* **105**, 010403 (2010).

35. J. L. Beckey, M. Cerezo, A. Sone, P. J. Coles, Variational quantum algorithm for estimating the quantum Fisher information. *Phys. Rev. Res.* **4**, 013083 (2022).

36. M. Cerezo, A. Sone, J. L. Beckey, P. J. Coles, Sub-quantum Fisher information. *Quantum Sci. Technol.* **6**, 035008 (2021).

37. M. Yu, D. Li, J. Wang, Y. Chu, P. Yang, M. Gong, N. Goldman, J. Cai, Experimental estimation of the quantum Fisher information from randomized measurements. *Phys. Rev. Res.* **3**, 043122 (2021).

38. I. Apellaniz, M. Kleinmann, O. Gühne, G. Tóth, Optimal witnessing of the quantum Fisher information with few measurements. *Phys. Rev. A* **95**, 032330 (2017).

39. A. Rath, C. Branciard, A. Minguzzi, B. Vermersch, Quantum Fisher information from randomized measurements. *Phys. Rev. Lett.* **127**, 260501 (2021).

40. V. Vitale, A. Rath, P. Jurcevic, A. Elben, C. Branciard, B. Vermersch, Robust estimation of the quantum Fisher information on a quantum processor. *PRX Quantum* **5**, 030338 (2024).

41. D.-J. Zhang, D. M. Tong, Krylov shadow tomography: Efficient estimation of quantum Fisher information. *Phys. Rev. Lett.* **134**, 110802 (2025).

42. Y.-H. Wang, D.-J. Zhang, Superiority of Krylov shadow tomography in estimating quantum Fisher information: From bounds to exactness. *npj Quantum Inf.* **12**, 74 (2026).

43. S. Aaronson, Shadow tomography of quantum states. *SIAM J. Comput.* **49**, STOC18-368–STOC18-394 (2020).

44. H.-Y. Huang, R. Kueng, J. Preskill, Predicting many properties of a quantum system from very few measurements. *Nat. Phys.* **16**, 1050–1057 (2020).

45. C. Hadfield, S. Bravyi, R. Raymond, A. Mezzacapo, Measurements of quantum Hamiltonians with locally-biased classical shadows. *Commun. Math. Phys.* **391**, 951–967 (2022).

46. S. Chen, W. Yu, P. Zeng, S. T. Flammia, Robust shadow estimation. *PRX Quantum* **2**, 030348 (2021).

47. T. Zhang, J. Sun, X.-X. Fang, X.-M. Zhang, X. Yuan, H. Lu, Experimental quantum state measurement with classical shadows. *Phys. Rev. Lett.* **127**, 200501 (2021).

48. Q.-C. Sun, Y.-F. Jiang, B. Bai, W. Zhang, H. Li, X. Jiang, J. Zhang, L. You, X. Chen, Z. Wang, Q. Zhang, J. Fan, J.-W. Pan, Experimental demonstration of non-bilocality with truly independent sources and strict locality constraints. *Nat. Photonics* **13**, 687–691 (2019).

49. Z.-D. Li, Y.-L. Mao, M. Weilenmann, A. Tavakoli, H. Chen, L. Feng, S.-J. Yang, M.-O. Renou, D. Trillo, T. P. Le, N. Gisin, A. Acín, M. Navascués, Z. Wang, J. Fan, Testing real quantum theory in an optical quantum network. *Phys. Rev. Lett.* **128**, 040402 (2022).

50. J.-W. Pan, M. Daniell, S. Gasparoni, G. Weihs, A. Zeilinger, Experimental demonstration of four-photon entanglement and high-fidelity teleportation. *Phys. Rev. Lett.* **86**, 4435–4438 (2001).

51. Y.-L. Mao, Z.-D. Li, S. Yu, J. Fan, Test of genuine multipartite nonlocality. *Phys. Rev. Lett.* **129**, 150401 (2022).

52. T. Zhang, W. Zhang, J.-T. Qiu, L. Huang, X. Yuan, Q.-M. Ding, Reliable quantum steering detection under imperfect measurement. *Phys. Rev. Lett.* **136**, 090201 (2026).

53. M. Abramowitz, I. A. Stegun, *Handbook of Mathematical Functions with Formulas, Graphs, and Mathematical Tables* (Dover Publications, New York, ed. 9, 1970), table 25.2.

54. M. Brenes, S. Pappalardi, J. Goold, A. Silva, Multipartite entanglement structure in the Eigenstate thermalization hypothesis. *Phys. Rev. Lett.* **124**, 040605 (2020).

55. X. Mi, P. Roushan, C. Quintana, S. Mandrà, J. Marshall, C. Neill, F. Arute, K. Arya, J. Atalaya, R. Babbush, J. C. Bardin, R. Barends, J. Basso, A. Bengtsson, S. Boixo, A. Bourassa, M. Broughton, B. B. Buckley, D. A. Buell, B. Burkett, N. Bushnell, Z. Chen, B. Chiaro, R. Collins, W. Courtney, S. Demura, A. R. Derk, A. Dunsworth, D. Eppens, C. Erickson, E. Farhi, A. G. Fowler, B. Foxen, C. Gidney, M. Giustina, J. A. Gross, M. P. Harrigan, S. D. Harrington, J.

Hilton, A. Ho, S. Hong, T. Huang, W. J. Huggins, L B Ioffe, S. V. Isakov, E. Jeffrey, Z. Jiang, C. Jones, D. Kafri, J. Kelly, S. Kim, A. Kitaev, P. V. Klimov, A. N. Korotkov, F. Kostritsa, D. Landhuis, P. Laptev, E. Lucero, O. Martin, J. R. McClean, T. M. Court, M. M. Ewen, A. Megrant, K. C. Miao, M. Mohseni, S. Montazeri, W. Mruczkiewicz, J. Mutus, O. Naaman, M. Neeley, M. Newman, M. Y. Niu, T. E. O'Brien, A. Opremcak, E. Ostby, B. Pato, A. Petukhov, N. Redd, N. C. Rubin, D. Sank, K. J. Satzinger, V. Shvarts, D. Strain, M. Szalay, M. D. Trevithick, B. Villalonga, T. White, Z. J. Yao, P. Yeh, A. Zalcman, H. Neven, I. Aleiner, K. Kechedzhi, V. Smelyanskiy, Y. Chen, Information scrambling in quantum circuits. *Science* **374**, 1479–1483 (2021).

56. A. Touil, S. Deffner, Information scrambling—A quantum thermodynamic perspective. *Europhys. Lett.* **146**, 48001 (2024).

57. W. Hoeffding, “A class of statistics with asymptotically normal distribution” in *Breakthroughs in Statistics: Foundations and Basic Theory* (Springer, 1992), pp. 308–334.

58. A. Rath, V. Vitale, S. Murciano, M. Votto, J. Dubail, R. Kueng, C. Branciard, P. Calabrese, B. Vermersch, Entanglement barrier and its symmetry resolution: Theory and experimental observation. *PRX Quantum* **4**, 010318 (2023).

59. B. Efron, Bootstrap methods: Another look at the Jackknife. *Ann. Statist.* **7**, 1–26 (1979).

60. S. N. Lahiri, *Resampling Methods for Dependent Data* (Springer, 2003).

61. E. Gilleland, Bootstrap methods for statistical inference. Part I: Comparative forecast verification for continuous variables. *J. Atmos. Oceanic Tech.* **37**, 2117–2134 (2020).

62. D. N. Politis, J. P. Romano, M. Wolf, *Subsampling* (Springer, 1999).

63. A. V. Rodionov, A. Veitia, R. Barends, J. Kelly, D. Sank, J. Wenner, J. M. Martinis, R. L. Kosut, A. N. Korotkov, Compressed sensing quantum process tomography for superconducting quantum gates. *Phys. Rev. B* **90**, 144504 (2014).

64. I. Strandberg, Simple, reliable, and noise-resilient continuous-variable quantum state tomography with convex optimization. *Phys. Rev. Appl.* **18**, 044041 (2022).

65. S. Mondal, A. K. Dutta, A modified least squares-based tomography with density matrix perturbation and linear entropy consideration along with performance analysis. *New J. Phys.* **b**, 083051 (2023).

66. A. Gaikwad, K. D. Arvind, True experimental reconstruction of quantum states and processes via convex optimization. *Quantum Inf. Process.* **20**, 19 (2021).

67. S. Diamond, S. Boyd, CVXPY: A python-embedded modeling language for convex optimization. *J. Mach. Learn. Res.* **17**, 2909–2913 (2016).

68. A. Gaikwad, M. S. Torres, S. Ahmed, A. F. Kockum, Gradient-descent methods for fast quantum state tomography. *Quantum Sci. Technol.* **10**, 045055 (2025).
